# {MFPP(R). An R package for matrix-based flexible project planning

**DOI:** 10.12688/f1000research.143144.1

**Published:** 2024-04-23

**Authors:** Zsolt Tibor Kosztyán, Aamir Saghir

**Affiliations:** 1 Department of Quantitative Methods, University of Pannonia, Veszprém, Veszprém, 8200, Hungary; 2 Department of Statistics, Mirpur University of Science and Technology, Mirpur, Khyber Pakhtunkhwa, Pakistan

**Keywords:** flexible projects, matrix-based planning, scheduling

## Abstract

Project planning and scheduling are essential parts of project management. While project planning and scheduling tools are already available to support traditional project management approaches, flexible project management, such as agile, extreme and hybrid project planning, are only somewhat supported by computer algorithms. To the best of our knowledge, no existing R package for project planning and scheduling can support project planning and scheduling for flexible projects. In this paper, the goal is to fill this gap; to this end, the R package mfpp for matrix-based flexible project planning/scheduling is introduced and described. This package includes a comprehensive set of tools for project managers to schedule both traditional and flexible project plans. The use of the package is illustrated through examples.

## Motivation and significance

Network planning methods are the main methods used to plan and schedule projects. ^1^ Among traditional methods, (a) the critical path method ^2^ and the metra potential method (or the precedence diagramming method) ^3^ can only handle tasks of a given duration, while (b) the program (or project) evaluation and review technique can handle stochastic activity durations, ^4^ while the graphical evaluation and review techniques can also handle decision events. ^5^ However, these methods were developed for the scheduling of traditional projects; consequently, for product or software development projects, they can be used only partially or not at all because these network planning methods cannot handle the specifics of these projects. ^6^^,^^7^ One of the main shortcomings of traditional network-based project planning techniques is that either the logic structure is static or a limited number of possible alternatives for completion sequences must be predefined. ^8^ In contrast, in cases of flexible project management, such as agile, extreme, or hybrid project management, the structure is not predefined. Instead, the real structure is determined by the decision of the customer-developer. In such flexible approaches, task completion is prioritized by customers’ claims. From the technological perspective, parallel and sequential completion can be allowed to handle flexible dependencies. ^9^ In addition, structural flexibility, which is a requirement in both the agile and extreme project management approaches is necessary to handle new non-planned tasks, while the hybrid project management approach requires that the features of the traditional, agile, and, if necessary, extreme project management approaches be combined. ^10^

In terms of scheduling, traditional time cost trade-off problems ^11^^,^^12^ support the traditional project management approach (TPMa) and are generally not or only minimally considered in the agile project management approach (APMa). In addition, other flexible approaches, such as the extreme project management approach (XPMa), allow for new unplanned tasks in response to changes in customer desires. The approach that has most recently begun to be explored is the hybrid project management approach (HPMa), which has a flexible structure but allows the application of traditional trade-off methods and/or multimode task completion (or alternative technologies). These approaches are detailed from the scheduling perspective in Table 1. ^13^

**Table 1.** Comparison of various traditional and flexible project management approaches [source: Ref. 13].

Approaches	Project structure	New tasks	Multiple modes
Traditional (TPMa)	Fixed	Not allowed	Handled
Agile (APMa)	Flexible	Not allowed	Not handled
Extreme (XPMa)	Flexible	allowed	Not handled
Hybrid (HPMa)	Flexible	Allowed	Handled

Flexible project planning approaches, such as APMa, HPMa, and XPMa, are very popular in software project planning. However, these approaches still lack algorithmic and software support for project scheduling. In answer to this, ^13^ developed the **mfpp** package in MATLAB (RRID:SCR_001622) to fill this gap, but this software is not free.

To the best of our knowledge, there are three packages in R (RRID:SCR_001905) available for project management. Among these, **PlotPrjNetworks** ^14^ and *plan* ^15^ are packages that create a Gantt diagram for the visualization of the project structure, while **ProjectManagement** ^16^ is a useful tool for managing a project from its development to its execution based on the TPMa. However, an R package that can manage a project based on APMa, XPMa, and HPMa is lacking. This is an important gap to fill for project planning/scheduling practitioners. Such a package would be useful to the user community because it could be integrated with other tools developed in R, meaning that it could be easily modified to suit the specific needs of each user and could be wrapped into a graphical interface.

Matrix-based techniques can be used instead of traditional network-based project planning techniques to model all types of changes in customer demands (such as new tasks and/or new subprojects) and parameters (such as time/cost/resource demands). Such methods have been successfully used to model agile projects. ^17^ The basis of a flexible matrix-based project planning method is a project domain matrix (PDM) ^17^ with unplanned tasks. The PDM is a matrix n+u by m+u , where n is the number of planned tasks, u is the number of unplanned tasks, m=n+w(3+ρ) , w is the number of possible completion modes and ρ is the number of possible resources. The PDM has five domains. The first domain is the logic domain (LD), which is described as an n(+u) by n(+u) project expert matrix (PEM), ^18^ a kind of numerical dependency structure matrix (NDSM). ^19^ The other domains are the time domain (TD) and the quality domain (QD), which are n(+u) by w submatrices, and the resource domain (RD), which is an n(+u) by w⋅ρ submatrix. The LD and QD contain real values between 0 and 1, while the TD, cost domain (CD), and RD contain nonnegative real values.

In this paper, we introduce **mfpp**, a new R package that provides the necessary tools to manage both traditional and flexible project plans. This package can be used to build matrix-based projects and calculate their demands, generate a flexible project network, and perform uncertainty analysis. The proposed package is an R version of the MATLAB functions (i.e. MATLAB functions from a previous study done by the author ^13^).

## Software description

**mfpp** is a new R package for project managers to schedule traditional and flexible project plans. In addition, it compares different project management approaches with respect to their scheduling performance and risk mitigation to help decision makers choose the best project management approach. Additionally, in the case of analyzing project libraries, scholars can analyze the various project management approaches and their resilience to risks. This package uses existing R packages **Matrix,** ^20^ **pracma** ^11^ and **Rfast** ^21^ to generate the PDM and calculate project values; the packages **genalg** ^22^ and **nsga2R** ^23^ for optimized resource allocation; and the **ggplot2** ^24^ and **igraph** ^25^ to plot the project structure. The **mfpp** package is available for download from CRAN and Code Ocean. ^26^ A summary of the functions incorporated into this package is provided in Table 2.

**Table 2.** Summary of functions in the mfpp package.

Function	Description
generatepdm	Generates the project domain matrix (PDM) for a flexible project planning problem
get.structures	Calculates the minimal/maximal/most likely project structures
is.flexible	Checks the flexibility of the project data matrix
maxscore_PEM	Calculates the maximal score value (PMAX) of possible project scenarios
minscore_PEM	Calculates the minimal score value of possible project scenarios
paretores	Calculates the Pareto-optimal resource allocation
Percent	Calculates the desired project completion characteristic of a project structure
phase1	Simulates estimation uncertainty
phase2	Simulates shock effects
phase3	Simulates the effects of a change in customer claims
plot.mfpp	Plotting function for matrix-based flexible project planning
summary.mfpp	Prints project data matrix constraints, matrices, lists, sets, etc.
Tpc	Calculates the cost demands of a project
Tpq	Calculates the total project quality for a project structure
Tpr	Calculates the maximum resource demands of a project
Tpt	Evaluates the activity times(early start time, early finish time, late start time and late finish time) of a project
truncpdm	Function for dropping excluded tasks

### Databases related to project management

Two project databases are directly included in this package. The first is the ^27^ database, which contains 240 simulated projects, all of which include four completion modes. This database can be used to test algorithms for the multimode resource-constrained project scheduling problem (MM-RC-PSP). The second database is provided by ^28^ and contains 125 real projects. These projects each include only one completion mode, but they also include the cost demands of the tasks.

## Use cases

The implementation of the R package **mfpp** is demonstrated using (i) simulated project structures and (ii) a real-life data set of project structures. The package must be loaded at the beginning of the session by typing the following:

install.packages("mfpp") *# Install mfpp package, if it is required*
library(mfpp)


### Simulated project structures

**Build fixed matrix-based projects and calculate their demands to support TPMa**

We start with the *tpt* function of the **mfpp** package to build fixed matrix-based projects and calculate their demands to support the TPMa. The LD specifies the structure of the project. The LD is an N-by-N (sub)matrix, where N is the number of tasks. The diagonal values represent the task priorities for task completion, where a value of 1 indicates a mandatory task, and a lower value indicates a supplementary task. The off-diagonal values represent the dependencies between tasks, where a value of 1 indicates a fixed dependency, and lower values indicate flexible dependencies between tasks. In the case of an acyclic graph, the LD can be reordered as an upper triangular matrix, which is assumed in this package. In the following example, a binary logic plan with one completion mode is specified. The *tpt* function determines the duration of the project (total project time, TPT) by calculating the task schedule, including time specifications such as the early start time (EST), early finish time (EFT), late start time (LST), late finish time (LFT), scheduled start time (SST), and scheduled finish time (SFT) for each task. The plot function draws a Gantt chart for ESTs, LSTs, or SSTs. The LD and TD must be specified as necessary and sufficient arguments for this function. The output of the function is as follows:

LD<-rbind(c(1,0,1), c(0,1,0), c(0,0,1))
colnames(LD)<-rownames(LD)<-paste("a",1:3,sep = "")
TD<-c(3,4,5)
TPT<-tpt(LD,TD)
summary(TPT)




##
##
##  Table of schedule
##    Dur EST EFT LST LFT TF SST SFT SF Is.Crit
## a1   3   0   3   0   3  0   0  3   0    TRUE
## a2   4   0   4   4   8  4   0  4   4   FALSE
## a3   5   3   8   3   8  0   3  8   0    TRUE




SST <- c(1,1,0)
TPT<-tpt(LD,TD,SST)
summary(TPT)




##
##
##  Table of schedule
##     Dur EST EFT LST LFT TF SST SFT  SF Is.Crit
##  a1   3   0   3   0   3  0   1   4  –1    TRUE
##  a2   4   0   4   4   8  4   1   5   3   FALSE
##  a3   5   3   8   3   8  0   4   9  –1    TRUE


**Figure 1.**
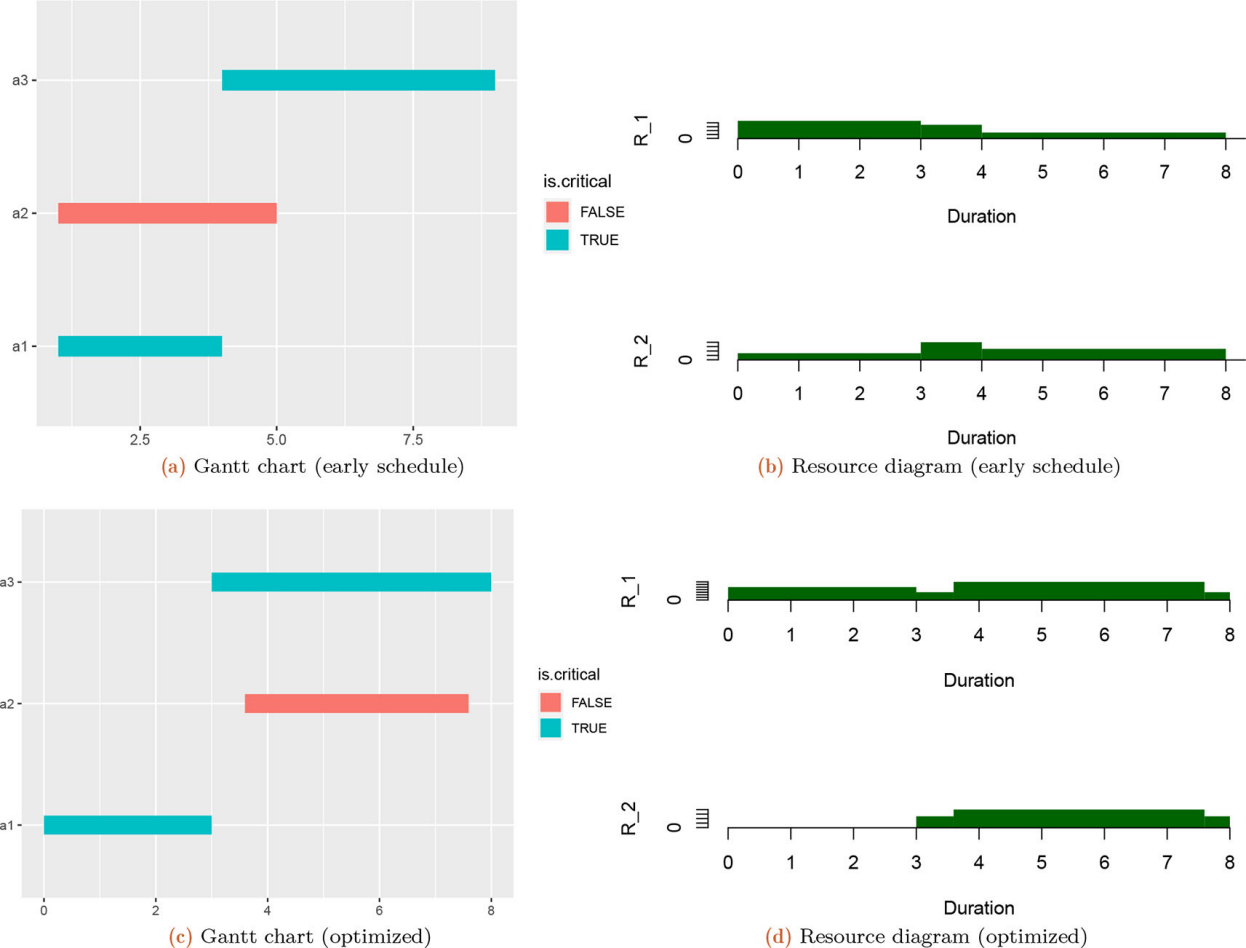
The scheduled Gantt chart and resource diagram for a binary logic plan with one completion mode. The total project resources based on two resources domains (R _1_ and R _2_) extracted through mfpp package.

The plot of the schedule can be drawn as follows ( Figure 1(a)):

Next, we illustrate the use of the *tpc*, *tpq* and *tpr* functions to calculate the total project cost, the total project quality, and the total project resources. The *tpr* function calculates the maximal resource demands and can specify a resource graph. In these functions, the CD and QD are N-by-w matrices, while the RD is an N-by- $\left(w^{*}r\right)$ matrix, where r is the number of resources. The CD is mandatory, while the QD and RD are optional matrices. The necessary arguments of these functions are the LD, CD, quality parameters (q), QD with completion modes, and RD. The output of this function for the given example is as follows:

set.seed(6)
CD<-c(10,20,24)
cat("\nTotal Project Cost (TPC): ",tpc(LD,CD))

##
## Total Project Cost (TPC): 54

q<-runif(3)
cat("\nTotal Project Quality (TPC): ",tpq(LD,LD,q))

##
## Total Project Quality (TPC):  0.5316529

QD2<-cbind(q,runif(3)) *# Generate two completion modes*
cat("\nRelative TPQ: ",tpq(LD,LD,q,QD2))

##
## Relative TPQ:   0.7169994

RD<-round(cbind(runif(3,min=0,max=5),runif(3,min=0,max=5)))
cat("\n\nTotal Project Resources (TPR)\n\n")

##
##
## Total Project Resources (TPR)

tpr(TPT$SST,LD,TD,RD)

##     R_1 R_2
## TPR   9   8


A plot of the total project resources can be called with the additional argument “res.graph=TRUe”, and the output is seen in Figure 1(b).

tpr(TPT$SST, LD, TD, res.graph = TRUE)

##     R_1 R_2
## TPR   9   8


Optimizing resource allocation is a combinatorial (NP-hard) problem; therefore, a metaheuristic method is used to minimize the maximum resource demands. In this case, the nondominated sorting genetic algorithm II (NSGA-II) method is used to minimize the maximum resource demands. In the case of multiple resources, a Pareto-optimal solution is found. The *paretores* function can help a project engineer determine the minimal resource demands while maintaining a specified project duration. The output of this function is as shown in Figure 1(c) and in Figure 1(d).

The set of domains, namely, the set of the LD, TD and CD and optionally the QD and RD specifies the PDM. A logic network can be constructed using this set of domains as follows:

PDM<-cbind(LD,TD,CD,q,RD)
class(PDM)<-"PDM_matrix"
summary(PDM,w=1,Rs=2)




##
## summary PDM matrix:
##    a1 a2 a3 TD  CD         q  
## a1  1  0  1  3  10 0.6062683 5 0
## a2  0  1  0  4  20 0.9376420 4 3
## a3  0  0  1  5  24 0.2643521 3 5
## attr(,"class")
## [1] "PDM_matrix"
##
## Minimal constraints:
##
## Summary of the PDM constraints structure:
##
## Time constraint (Ct): 8
## Const constraint (Cc): 54
## Score/scope constraint (Cs):  1
## Quality constraint (Cq):  0.5316529
##
## Maximal constraints:
##
## Summary of the PDM constraints structure:
##
## Time constraint (Ct): 8
## Const constraint (Cc): 54
## Score/scope constraint (Cs): 1
## Quality constraint (Cq): 0.5316529
## Resource constraint(s) (CR):
##     R_1 R_2
## TPR   9   8


**Generation of a flexible project network to support the HPMa**

The HPMa combines the features of the TPMa (multiple completion modes), APMa (a flexible project structure), and XPMa (new, unplanned tasks). Project plans to support the HPMa can also be designed using the proposed **mfpp** package. For this purpose, the first function that is needed is *generatepdm.* The input arguments of this function are the number of tasks (N); the flexibility factor (ff); the connectivity factor (cf); the maximum values of the TD (mTD), CD (mCD), RD (mRD); the number of modes (w); the number of resources (nR); the number of possible extra tasks (nW); and the scale and QD of the project scenario. The function returns either the PDM only or a PDM list that also contains the number of completion modes (w) and the number of resources (Rs). Consider a flexible plan with 4 planned tasks, 2 completion modes and 2 resources. The project schedule generated using the HPMa is as follows:

*# Generation of PDM matrix for flexible project planning MFPP package.*

*# Define number of modes, flexibility factor and connectivity factor of a project scenario.*
N=5;ff=0.30;cf=0

*# Define maximum value of time domain, cost domain and resource domain of a project scenario.*
mTD=3;mCD=4;mRD=3

*# Define number of modes, number of resources, number of possible extra tasks (nW), scale and# quality domain of a project scenario.*

w=2;nR=2;nW=1
scale=1.6
PDM<-generatepdm(N,ff,cf,mTD,mCD,mRD,w,nR,nW,scale,QD=TRUE,lst=TRUE)
rownames(PDM$PDM)<-colnames(PDM$PDM)[1:(N+nW)]<-paste("a",1:(N+nW),sep="")

summary(PDM) *# Summary of PDM list*




##
## summary PDM list:
##
## Number of completion modes (w): 2
## Number of resources (Rs): 2
## summary PDM matrix:
##   a1        a2        a3        a4 a5 a6       t_1      t_2       c_1
## a1 1 0.6348251 0.0000000 1.0000000  0  0 1.8922066 2.206381 2.9384623
## a2 0 1.0000000 1.0000000 0.0000000  0  0 0.1397774 0.160757 0.5917513
## a3 0 0.0000000 0.9458257 0.0000000  0  0 2.7119461 2.784497 3.1012793
## a4 0 0.0000000 0.0000000 0.7420972  0  0 1.8202474 1.896929 0.5478731
## a5 0 0.0000000 0.0000000 0.0000000  1  0 2.6490032 2.922064 2.3434704
## a6 0 0.0000000 0.0000000 0.0000000  0  0 0.0000000 0.000000 0.0000000
##          c_2       q_1       q_2     r_1.1    r_2.1     r_1.2     r_2.2
## a1 2.9456676 0.4591163 0.4597432 1.7602166 1.767857 0.7749634 0.8299197  
## a2 0.7051308 0.2476199 0.2898660 0.9645262 1.109172 1.9847076 2.0104963  
## a3 3.8735180 0.3979600 0.4327975 2.3338386 2.723688 2.3193125 2.3997095  
## a4 0.6072272 0.2989701 0.3343502 1.8691629 2.192587 2.5611961 2.9716221  
## a5 2.5560046 0.4891549 0.5480301 2.2484773 2.280209 2.2384662 2.4961772  
## a6 0.0000000 0.0000000 0.0000000 0.0000000 0.000000 0.0000000 0.0000000  
## attr(,"class")
## [1] "PDM_matrix"
##
## Minimal constraints:
##
## Summary of the PDM constraints structure:
##
## Time constraint (Ct): 2.711946
## Const constraint (Cc): 5.873684
## Score/scope constraint (Cs): 0.4907664
## Quality constraint (Cq): 0.1039251
##
## Maximal constraints:
##
## Summary of the PDM constraints structure:
##
## Time constraint (Ct): 2.945254
## Const constraint (Cc): 10.68755
## Score/scope constraint (Cs): 0.9427112
## Quality constraint (Cq): 0.3830448
## Resource constraint(s) (CR):




##          R_1      R_2
## TPR 7.196484 7.867509


The main components of flexible project structures can be drawn using the *plot* function of the **mfpp** package, where these structures are defined as follows.

**Minimal structure:** This structure contains only mandatory tasks and fixed dependencies. It provides the lowest quality, cost, and time demands when the lowest quality, lowest cost, and shortest completion modes are specified for each task.

**Maximal structure:** This structure contains both mandatory and supplementary tasks as well as both fixed and flexible dependencies. It provides the highest quality, greatest cost, and greatest time demands when the highest quality, greatest cost, and longest completion modes are specified for each task.

**Minimax structure:** This structure contains only mandatory tasks but both fixed and flexible dependencies. It can provide the lowest resource demands.

**Maximin structure:** This structure contains both mandatory and supplementary tasks, but only fixed dependencies. It can provide the greatest resource demands.

**Figure 2.**
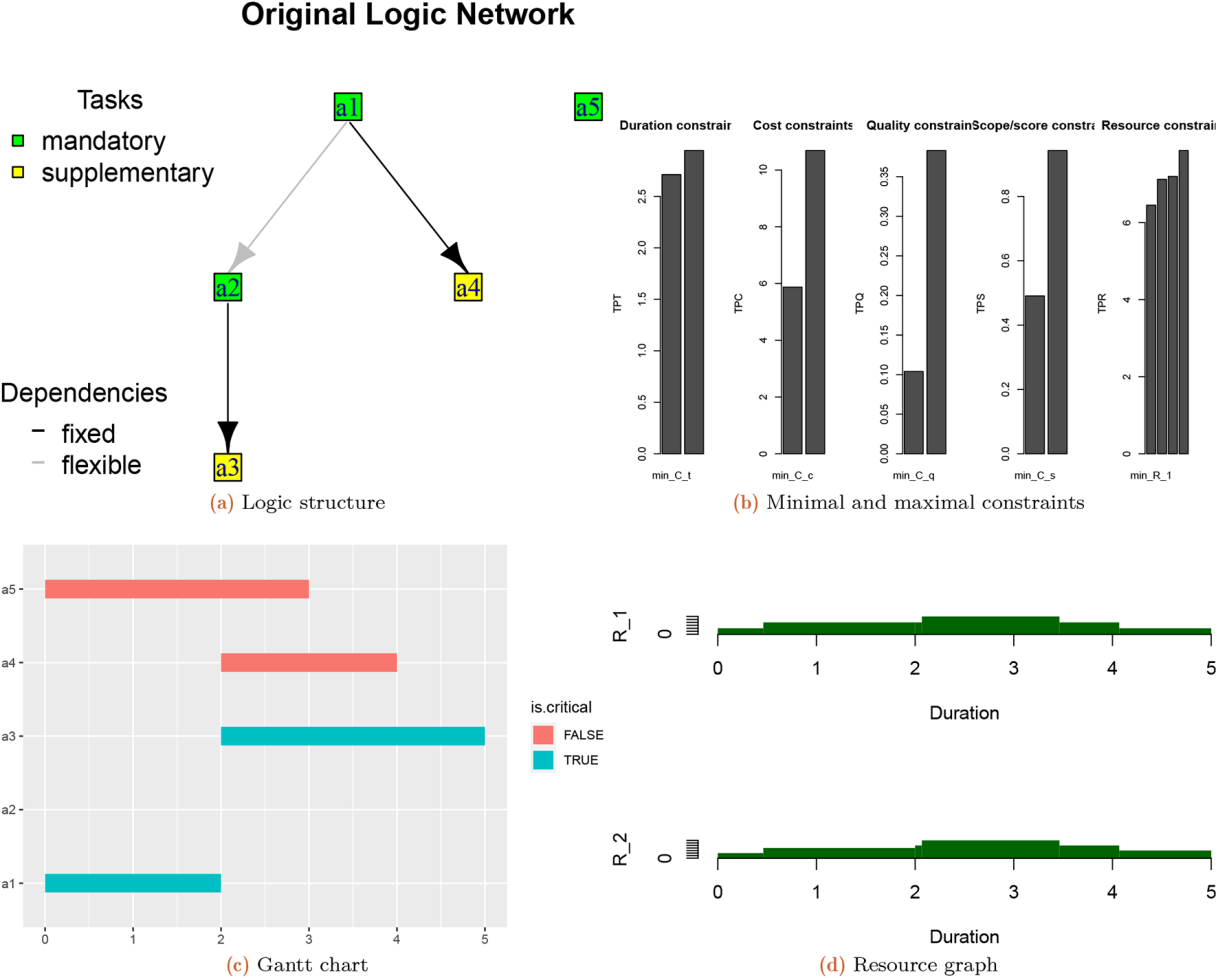
Logic structure (a), minimal and maximal constraints (b) of the hybrid-project; Gantt chart (c) and Resource graph (d) of optimal schedule for completion mode 1.

**Most-likely structure:** Rounding the values of the LD yields the most-likely structure. The plot of the PDM structure is as follows in Figure 2(a):

The plot of the constraints is as follows in Figure 2(b). The most likely/most desired structures of the project for multiple modes can be determined using **mfpp**. The input and output are as follows:

PSM_list<-get.structures(PDM,type = "most")$moststruct
summary(PSM_list)




##
## summary PDM list:
##
## Number of completion modes (w):  2
## Number of resources (Rs):  2
## summary PDM matrix:
##   a1 a2 a3 a4 a5 a6       t_1      t_2       c_1       c_2       q_1       q_2
## a1 1  1  0  1  0  0 1.8922066 2.206381 2.9384623 2.9456676 0.4591163 0.4597432
## a2 0  1  1  0  0  0 0.1397774 0.160757 0.5917513 0.7051308 0.2476199 0.2898660
## a3 0  0  1  0  0  0 2.7119461 2.784497 3.1012793 3.8735180 0.3979600 0.4327975
## a4 0  0  0  1  0  0 1.8202474 1.896929 0.5478731 0.6072272 0.2989701 0.3343502
## a5 0  0  0  0  1  0 2.6490032 2.922064 2.3434704 2.5560046 0.4891549 0.5480301
## a6 0  0  0  0  0  0 0.0000000 0.000000 0.0000000 0.0000000 0.0000000 0.0000000
##        r_1.1    r_2.1     r_1.2     r_2.2 
## a1 1.7602166 1.767857 0.7749634 0.8299197
## a2 0.9645262 1.109172 1.9847076 2.0104963
## a3 2.3338386 2.723688 2.3193125 2.3997095
## a4 1.8691629 2.192587 2.5611961 2.9716221
## a5 2.2484773 2.280209 2.2384662 2.4961772
## a6 0.0000000 0.000000 0.0000000 0.0000000
## attr(,"class")
## [1] "PDM_matrix"
##
## Minimal constraints:
##
## Summary of the PDM constraints structure:
##
## Time constraint (Ct): 2.851723
## Const constraint (Cc): 9.522836
## Score/scope constraint (Cs): 1
## Quality constraint (Cq): 0.3665425
##
## Maximal constraints:
##
## Summary of the PDM constraints structure:
##
## Time constraint (Ct): 2.945254
## Const constraint (Cc): 10.68755
## Score/scope constraint (Cs): 1
## Quality constraint (Cq): 0.4025323
## Resource constraint(s) (CR):




##          R_1      R_2
## TPR 7.196484 7.867509


The optimal schedules for different completion modes can be calculated using the *paretores* and *truncpdm* functions by dropping excluded tasks and their demands. The optimal resource allocation can be calculated once the completion modes are selected for each task as follows:

*# Get PSM matrix*

*# Drop excluded tasks, and their demands*

PSM<-round(truncpdm(PSM_list$PDM))

w<-PSM_list$w *# Get number of completion modes*
Rs<-PSM_list$Rs *# Get number of resurces*

DSM<-PSM[,1:nrow(PSM)] *# Get PSM matrix*

*# Get time demands (time domain, TD)*
TD<-PSM[,paste("t",1:w,sep = "_")]

*# Get resource demands (resource domain, RD)*
RD<-PSM[,tail(colnames(PSM),w*Rs)]

*# Calculate optimal schedules for both completion modes*

RES1<-paretores(DSM,TD[,1],RD[,1:Rs])
RES2<-paretores(DSM,TD[,2],RD[,(Rs+1):(2*Rs)])


Figure 2(b) shows the Gantt chart and Figure 2(c) shows the resource graph of the optimal schedule for the completion mode 1.

## Project structure database

In this section, Boctor’s publicly available ^27^ simulation database is used to demonstrate the applicability of **mfpp**. This database contains a collection of 240 projects with different names, numbers of completion modes (w) and numbers of resources (Rs). The details of the database can be retrieved using the summary function. However, we are using **project number 2** within this database, whose details are as follows: name of project= boct10, w= 4 and Rs= 2. Any other project could also be used to obtain the optimal project structure and resources.

### Obtaining a project structure from the collection

**Figure 3.**
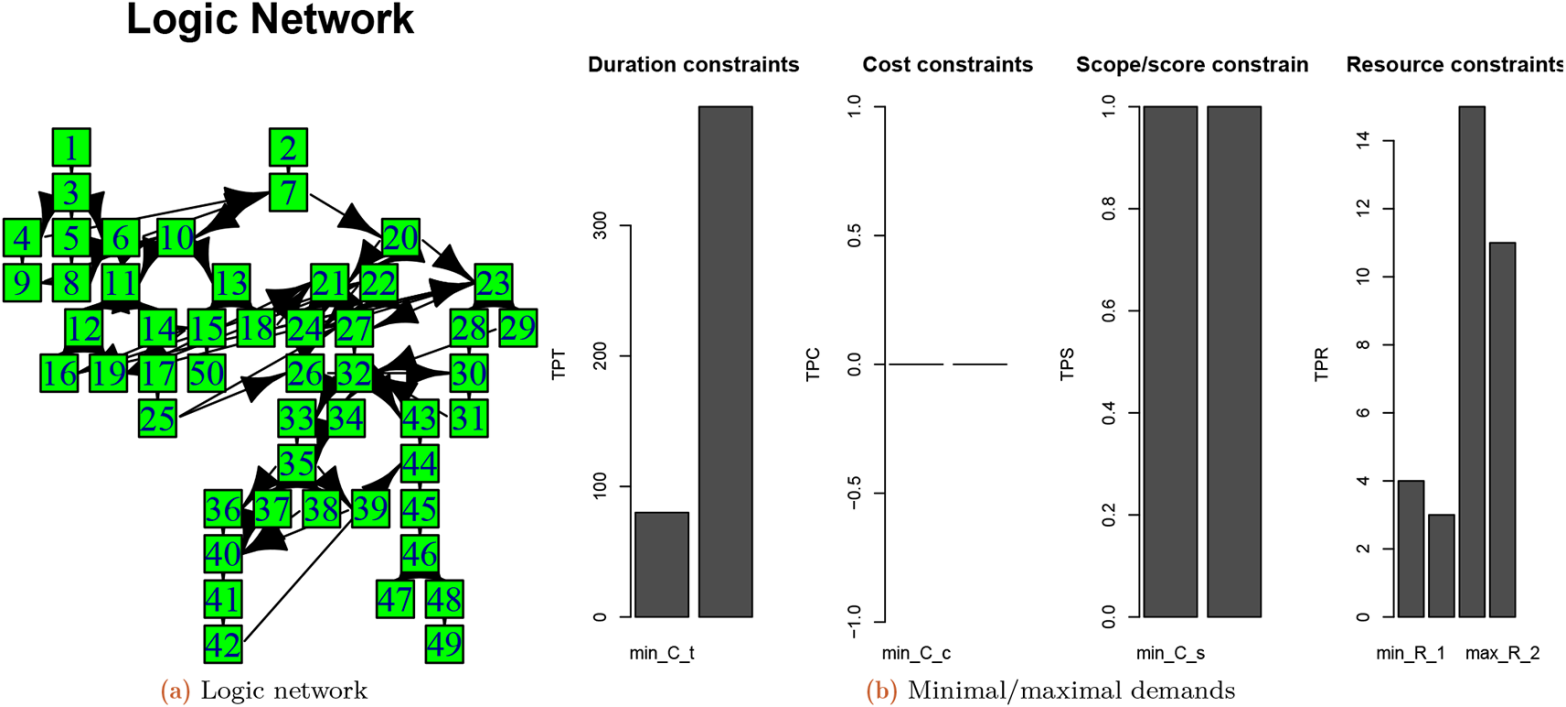
Logic network and minimal/maximal demands of project based on 4 completion mode using the Boctor database.

Figure 3(a) shows the original project structure and Figure 3(b) shows the minimal/maximal constraints of a project 2 from the Ref. 27’s project collection.

**Figure 4.**
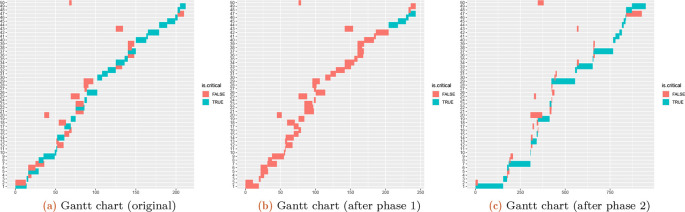
Gantt chart of the selected project for the 1st completion mode scheduled by early starting time (a); after varying project demands (b-c) (see phase 1 and phase 2).

The Gantt charts of the selected project of completion mode 1 is constructed by extracting the PDM and TD as follows (see Figure 4(a)):

### Sensitivity analysis

For project risk analysis, one of the most frequently used methods is sensitivity analysis. The sensitivity analysis procedure in **mfpp** consists of three phases. **Phase 1:** Effects of uncertainty **Phase 2:** Effects of shocks **Phase 3:** Structural changes.

Notably, phase 1 and phase 2 involve varying the demands, while phase 3 varies the project structure. Phase 1 affects the entire project, while phase 2 and phase 3 affect only selected demands or structural elements. These phases can be performed in a sequential or parallel manner. The phases follow the logic of. ^10^ The **mfpp** package also analyzes the effects of uncertainty on the scheduled project using the *phase1* and *phase2* functions. Furthermore, the existing project plan can be modified to a flexible structure using the *phase3* function of the developed package. These functions are also demonstrated on the selected project from the database.

**Effects of uncertainty**

Phase 1 serves to analyze the uncertainty of the estimation. Modified demands are generated in the interval of ([o+a,o+b]) , where o is the original value. The random generator can be specified to follow either a *uniform* (=default) or *beta* distribution. These modifications are applied to all kinds of demands for each task (compare Figures 4(a), 4(b), and 4(c)).

**Effects of shocks**

Phase 2 simulates shock effects, by increasing p percent of the task demands by a factor of up to s . Phase 2 investigates the effects of shocks, in which not all task demands are changed but the changes are significant (see Figure 4(b)).

**Structural changes**

**Figure 5.**
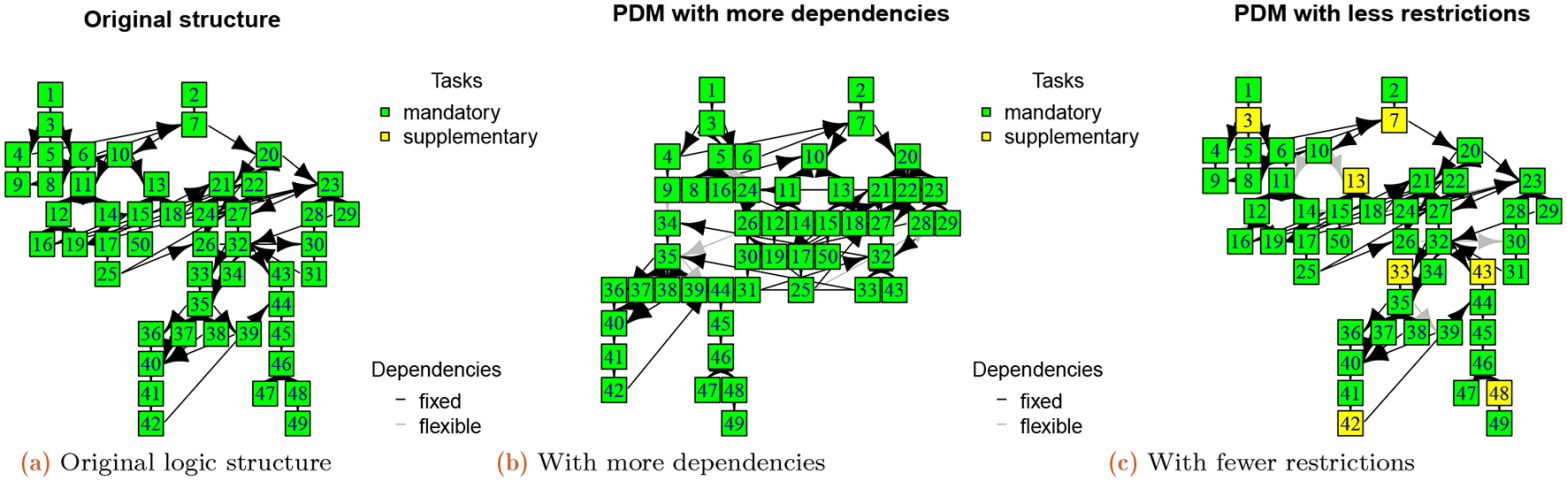
Logic network of the original structure (a), with more dependencies (b), and with fewer restrictions (c) (see phase 3 function).

In phase 3, P percent of the nodes ( *i.e.,* tasks) or arcs ( *i.e.,* dependencies)are selected to change the corresponding scores by up to the maximal change effect S . Phase 3 is simulates changes in customer priorities and technological changes. In the case of S<0 , the plan will be more flexible, whereas S>0 increases priorities and can produce new dependencies (compare Figures 5(a), 5(b), and 5(c)).

## Conclusions

The **mfpp** package has been developed to provide users with a comprehensive set of functions that can be used to create matrix-based models for both traditional and flexible project management approaches. The presented package also compares different project management approaches with respect to their scheduling performance and risk mitigation to help decision-makers choose the best project management approach. Moreover, in the case of analyzing project libraries, scholars can analyze various project management approaches and their resilience to risks. The **mfpp** tool can help decision-makers determine which approaches are best for their requirements.

## Data Availability

The mfpp package includes matrix-based version of publically available Boctor’s ^27^ and Batseliers’s ^28^ project datesets.

## Software availability

**Source code available from:** https://codeocean.com/capsule/5196188/tree/v1

**Archived source code at the time of publication:** https://doi.org/10.24433/CO.2915306.v1 ^26^

**License:** MIT license

mfpp can be installed via CRAN using install.packages(“mfpp”).

mfpp is maintained at http://github.com/kzst/mfpp.
