# {MFPP(R). An R package for matrix-based flexible project planning

**DOI:** 10.12688/f1000research.143144.2

**Published:** 2024-09-12

**Authors:** Zsolt Tibor Kosztyán, Aamir Saghir

**Affiliations:** 1 Department of Quantitative Methods, University of Pannonia, Veszprém, Veszprém, 8200, Hungary; 2 Department of Statistics, Mirpur University of Science and Technology, Mirpur, Khyber Pakhtunkhwa, Pakistan

**Keywords:** flexible projects, matrix-based planning, scheduling

## Abstract

Project planning and scheduling are essential parts of project management. While project planning and scheduling tools are already available to support traditional project management approaches, flexible project management approaches, such as agile, extreme, and hybrid project planning, are less well supported by software tools, especially freely available software packages. To our knowledge, no existing R package for project planning and scheduling can support flexible projects. This paper aims to fill this gap by introducing and describing the R package mfpp for matrix-based flexible project planning/scheduling. This package includes a comprehensive set of tools for project managers to schedule both traditional and flexible project plans. The use of the package is illustrated through examples.

## Motivation and significance

Project planning and scheduling algorithms have been intensively researched during the past 60 years ^1^; however, with a few exceptions (such as the examples in Refs. 2– 4), these algorithms assume fixed dependencies between tasks and neglect the task completion priority. Since traditional network-based project planning techniques either consider logic structures to be static or require a limited number of possible alternatives for completion sequences to be predefined, ^2^ they are very difficult to use to support flexible projects, such as agile, hybrid, or extreme projects. ^1^^,^^5^ In flexible project management, the completion structure is not predefined. Instead, the real structure is determined by the decision of the customer–developer. In such flexible approaches, task completion is prioritized according to customers’ claims. From a technological perspective, parallel and sequential completion can be used to handle flexible dependencies. ^6^ In addition, structural flexibility, which is a requirement of both agile and extreme project management approaches, is necessary to handle new unplanned tasks, whereas the hybrid project management approach requires combining the features of traditional, agile, and, if necessary, extreme project management approaches. ^5^ Although researchers currently focus mainly on scheduling traditional projects, flexible project planning techniques have become more popular in recent decades, especially in software project scheduling [see the results of surveys in Refs. 7, 8].

Flexibility in project scheduling refers to the ability to make scheduling decisions. There are multiple categories of flexibility ^9^: *Time-related flexibility* in a project plan is achieved through the use of slacks or topological floats. These allow for changes in the scheduled start and finish times of tasks without affecting the order in which tasks need to be completed or the way they are carried out. This method can change the duration of tasks by establishing minimum and maximum time delays. *Activity or modal flexibility* refers to the ability to execute a task in many modes or with distinct sets of tasks using diverse combinations of resources. This is represented via Petri nets, required and optional choices, or AND/OR networks, which expand resource-constrained project scheduling problems by including alternate activity chains. Activity or modal flexibility is used in nondeterministic methods, such as discrete/continuous/stochastic-time-cost methods and time-quality-cost tradeoff project scheduling techniques. ^10^ Traditional and hybrid project management approaches usually employ these techniques for resource-constrained project scheduling problems. ^10^^,^^11^ *Dependency flexibility* allows some logical dependencies to be omitted, which enables tasks to be executed concurrently or in any desired sequence. *Scope flexibility* allows for the exclusion or delay of low-priority tasks, which reduces the need for resources and may result in a shorter project time at the expense of quality or level of completion. *Structural flexibility*, which describes the combined effects of dependency and scope flexibility, has a significant effect on the logical structure of agile projects and extreme projects. ^9^ This method can be used to calculate all types of flexibility, but when the effects of flexibility are investigated, we focus on structural flexibility; therefore, we usually omit the structural term.

Advanced project management tools such as Microsoft Project, JIRA, Wrike, Teamwork, Zoho Projects, ^12^ Asana, ^13^ and Monday.com ^14^ are backed by highly sophisticated algorithms and are widely used to manage adaptable projects for numerous multinational companies. Nevertheless, the target audience of these software applications is professionals and generally not scholars. These software applications are not freely available. The algorithms for managing flexible projects with these applications are hidden from the user. In general, they provide only the most likely solution and not all possible solutions; therefore, they do not sufficiently support adaptability. By implementing the algorithm of Kosztyán and Szalkai, ^4^ all possible solutions, including schedules with minimal time, cost, and resource demands, can be obtained. Since the proposed freely available package is implemented in R, other statistical and analysis tools can be integrated to investigate the nature of flexible projects. In this way, scholars can gain better insight into scheduling flexible projects.

In addition, while most studies and state-of-the-art project management tools focus only on the risk of changing project demands, the proposed mfpp package, following Kosztyán et al., ^5^ allows us to analyze the shock effects for project demands and the effects of structural changes. This extension is essential for analyzing the risk effects of flexible projects.

The suggested R package for matrix-based flexible project planning offers numerous potential contributions to the current body of knowledge on project management and project tools.

• **The integration of flexible approaches**: This package incorporates elements from classic, agile, extreme, and hybrid project management processes. The comprehensive solutions provided enable a diverse range of project types and management styles. This integration can improve understanding of how diverse techniques can coexist and be implemented in practical situations.
• **Algorithm transparency**: In contrast to most commercial project management applications that employ proprietary algorithms, the mfpp package provides transparency by utilizing the algorithm of Kosztyán and Szalkai. ^4^ This algorithm enables users to examine all potential scenarios for project scheduling. This transparency can enhance scholarly investigations and real-world implementations by enabling a valuable understanding of the fundamental mechanisms of adaptable project scheduling.
• The package **provides assistance for managing unforeseen tasks** and modifications of the project’s scope, emphasizing the importance of adaptability in project management. The emphasis on flexibility in the project management literature is in line with contemporary trends that highlight the importance of sensitivity to change.
• The mfpp package, built in R, enables the **integration** of many statistical and analytical methods. This can facilitate more advanced analyses of project data, thereby improving the empirical basis of research in project management.
• The mfpp package enables the **execution of empirical studies** to assess the efficacy of flexible project planning methodologies. This contributes to the existing body of knowledge by offering data-driven insights and case studies that support theoretical models.
• **Enhanced risk analysis**: The mfpp package enables the analysis of changes in not only task and project demands but also completion scores; therefore, the effects of structural changes can also be analyzed. To demonstrate the impact of shocks, the package can randomly select activities whose demand changes to a large extent (shockwise).
• **Educational resource**: The mfpp package can be used as a publicly accessible tool for researchers and practitioners. It can serve as an educational resource by assisting in training future project managers in flexible planning approaches and the utilization of R for project management.

## Scheduling flexible projects

In terms of scheduling, traditional time–cost trade-off problems ^10^^,^^15^ support the traditional project management approach (TPMa) and are generally not considered or are only minimally considered in the agile project management approach (APMa). Other flexible approaches, such as the extreme project management approach (XPMa), allow for new unplanned tasks in response to changes in customer desires. Most recently, the hybrid project management approach (HPMa) has begun to be explored; it has a flexible structure but allows the application of traditional trade-off methods and/or multimode task completion (or alternative technologies). These approaches are detailed from a scheduling perspective in Table 1. ^16^

**Table 1.** Comparison of various traditional and flexible project management approaches [source: Ref. 16].

Approach	Project structure	New tasks	Multiple modes
Traditional (TPMa)	Fixed	Not allowed	Handled
Agile (APMa)	Flexible	Not allowed	Not handled
Extreme (XPMa)	Flexible	allowed	Not handled
Hybrid (HPMa)	Flexible	Allowed	Handled

Flexible project planning approaches, such as APMa, HPMa, and XPMa, are very popular in software project planning. However, these approaches still lack algorithmic and software support for project scheduling. To address this, ^16^ developed the **mfpp** package in MATLAB (RRID:SCR_001622) to fill this gap, but this software is not free.

To our knowledge, there are three packages available in R (RRID:SCR_001905) for project management. Among these, **PlotPrjNetworks** ^17^ and *plan* ^18^ create a Gantt diagram for the visualization of the project structure, whereas **ProjectManagement** ^19^ is a useful tool for managing a project from development to execution that is based on the TPMa. However, an R package for project management that is based on APMa, XPMa, and HPMa is lacking. This is an important gap to fill for project planning/scheduling practitioners. Such a package would be useful to the user community because it could be integrated with other tools developed in R, meaning that it could be easily modified to suit the specific needs of each user and could be wrapped into a graphical interface.

## Brief summary of matrix-based project planning and risk analysis

Matrix-based techniques can be used instead of traditional network-based project planning techniques to model all types of changes in customer demands (such as new tasks and/or new subprojects) and parameters (such as time/cost/resource demands). Such methods have been successfully used to model agile projects. ^3^ The basis of a flexible matrix-based project planning method is a project domain matrix (PDM) ^3^ with unplanned tasks. The PDM is a matrix of dimensions n+u by m+u , where n is the number of planned tasks, u is the number of unplanned tasks, m=n+w(3+ρ) , w is the number of possible completion modes and ρ is the number of possible resources. The PDM has five domains. The first domain is the logic domain (LD), which is described as an n(+u) by n(+u) project expert matrix (PEM), ^20^ a type of numerical dependency structure matrix (NDSM). ^21^ The other domains are the time domain (TD) and the quality domain (QD), which are n(+u) by w submatrices, and the resource domain (RD), which is an n(+u) by w⋅ρ submatrix. The LD and QD contain real values between 0 and 1, whereas the TD, cost domain (CD), and RD contain nonnegative real values.

The diagonal values in the LD matrix encode the completion priorities. A value of 1 indicates *mandatory tasks.* These tasks cannot be excluded from the project or postponed. In TPMa, all diagonal values are considered 1. If a diagonal value is $0<{\left[\mathrm{LD}\right]}_{\mathrm{ii}}=l_{\mathrm{ii}}<1$ , the corresponding task is called a *supplementary task*, which means that completion is flexible. On the basis of the priority and the time/cost/resource demands, the task will either be included in the project or postponed. Notably, if the activity is excluded from the project plan, all the dependencies and needs of the activity are deleted from the project plan (or postponed to the next (sub)project). The out-diagonal values in the LD domain indicate dependencies. $l_{\mathrm{ij}}=1$ ( $l_{\mathrm{ij}}=0,i\neq j$ ) indicates *fixed dependency* ( *no dependency*) between task i and task j . Fixed dependency means that the start of task j must wait until the end of task i . In flexible projects, $0<l_{\mathrm{ij}}<1$ indicates *flexible dependency*, which means that on the basis of the constraints and the target functions, the task can be included or excluded from the project. The prescription of the dependency indicates serial completion, whereas resolving the dependency allows parallel completion of the tasks. TPMa allows only mandatory tasks and fixed dependencies; therefore, ∀i,j $l_{\mathrm{ij}}\in \left\{0,1\right\}$ . APMa, HPMa, and XPMa allow flexible dependencies; therefore, ∀i,j $l_{\mathrm{ij}}\in \left\{0,1\right\}$ .

TPMa and HPMa allow multiple *completion modes.* This means that a task i can be completed in w ways. Each completion mode (called a technology) has time, cost, quality and resource demands. The optimization algorithm must select the appropriate technologies for each task to fit the constraints and achieve the best target value.

XPMa and HPMa allow us to consider new tasks. This means that nW new columns in the LD domain and nW new rows can be considered for each domain.

**Figure 1.**
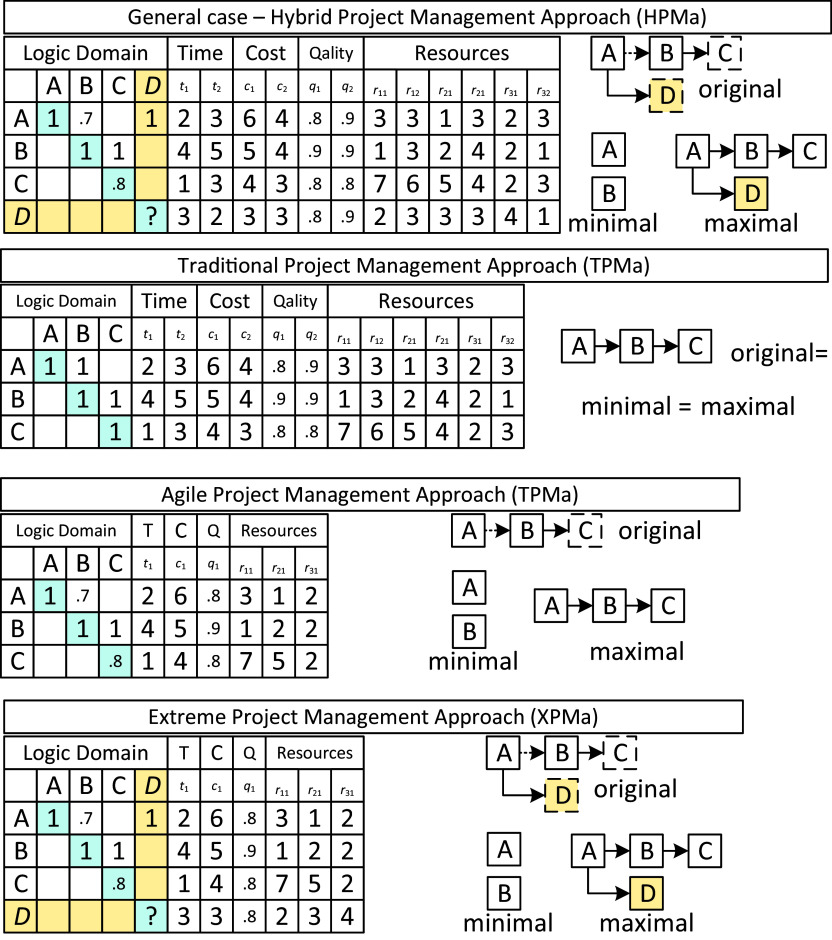
An example for comparing initial matrices for different kinds of project management approaches.

Figure 1 compares the initial matrices of the various project management approaches.

The example involves two mandatory tasks (A, B), one supplementary task (C) and one nonplanned task (D). APMa and XPMa consider only one completion mode (usually the first), whereas HPMa (the general case) and XPMa consider two different completion modes (technologies). Since TPMa does not use completion scores, it does not distinguish between fixed and flexible dependencies or between mandatory and supplementary tasks. However, TPMa requires replanning if nonplanned tasks occur.

### Demands

Kosztyán and Szalkai ^4^ showed that the following demands can be calculated.

The total project time (TPT) is the makespan of the project durations of tasks that are to be completed for selected completion modes. The minimal (maximal) value of TPT occurs if only mandatory tasks (both mandatory and supplementary tasks) and fixed (fixed and flexible) dependencies are included in the project, and technologies are selected so that the possible duration is minimal (maximal).

The total project cost (TPC) is the cost of deciding to complete the tasks. The TPC is minimal (maximal) if only mandatory (all) tasks are included in the project with the minimal (maximal) possible cost.

The total project quality (TPQ) value is the sum of the quality scores of the completed tasks. The TPQ is minimal (maximal) if only mandatory (all) tasks are included in the project with the minimal (maximal) possible quality score value.

The amount of total project resources (TPR) is the maximal resource demand for the tasks that are to be completed. The TPR is minimal (maximal) if only mandatory (all) tasks are included, but all (only fixed) dependencies are included in the project plan with minimal (maximal) possible resource demands.

The total project score (TPS) is the product of the scores of tasks that are to be included and the scores of tasks that are to be excluded. Kosztyán ^3^ suggested that the score of exclusion should be one minus the score of inclusion, but this is not necessary. The TPS is minimal (maximal) if all the mandatory and supplementary tasks are included, where the score of exclusion is higher (lower) than the score of inclusion. If the TPS is maximal, we say that this solution is the *most desired* solution.

### Relative constraints and target functions

Since minimal and maximal values of demands can be calculated, the relative values of the constraints can be specified in the following way:

$$C_{x}\%=\frac{C_{x}-{\text{TPX}}_{\_\text{max}}}{{\text{TPX}}_{\_\text{min}}-{\text{TPX}}_{\_\text{max}}}$$ (1)

where $C_{X}$ is the constraint of demand X ( X can be time, cost, resource demand or quality or score value). ${\text{TPX}}_{\text{min}}$ ( ${\text{TPX}}_{\text{max}}$ ) is the minimal (maximal) value of demand X .

The target functions can be described as follows:

min←TPT(PDM) (2)

min←TPC(PDM) (3)

min←TPR(PDM) (4)

max←TPQ(PDM) (5)

max←TPS(PDM) (6)

If all target functions must be considered, we can either specify a composite function or find the Pareto optimal function for these target functions.

The optimal solution can be found via a genetic algorithm, ^4^^,^^16^ where a chromosome encodes a possible solution. A chromosome has three parts. The first part encodes the supplementary tasks and flexible dependencies. These can be either 0 or 1 to indicate exclusion (0) or inclusion (1). The second part uses integer values, which encode the selected completion modes. The last part encodes the scheduled start times.

The target functions are the same in each project management approach, as we mentioned previously, and only the structure of the PDM matrix is different.

In this paper, we introduce **mfpp**, a new R package that provides the necessary tools to manage both traditional and flexible project plans. This package can be used to build matrix-based projects and calculate their demands, generate a flexible project network, and perform uncertainty analysis. The proposed package is an R version of MATLAB functions ( *i.e.*, MATLAB functions from the previous study Kosztyán ^16^).

### Supporting risk analysis

With the proposed package, three kinds of risks can be generated. The first (phase 1) supports traditional risk analysis. In this way, task (time/cost/resource) demands can be changed with a unified β distribution.

The second (phase 2) function simulates the shock effect. A predefined percentage of tasks are selected, and their demands are changed significantly, but other task demands remain unchanged. This function can simulate shock effects when the change is limited to one task ( *e.g.*, the running task), but these effects may also be long-lasting ( *e.g.*, a virus attack).

The third (phase 3) function can change the task priorities and scores of flexible dependencies. Changes in task priorities simulate changes in customer needs, and changes in dependencies simulate changes in technological needs.

## Experiments

In this section, we examine the analytical possibilities of flexible project planning methods through generated, synthetic (Boctor ^22^) and real (Batselier and Vanhoucke ^23^) project databases. The aims of this section are threefold. First, the types of investigations that scholars can use to analyze flexible project structures are demonstrated. Second, it is shown how different parameters, such as the number of resources, number of tasks, number of completion methods, and percentage of (structural) flexibility, affect the reduction in project duration. Third, the effects on duration reduction achieved by different project management approaches are compared. If the number of tasks or relationships that can be omitted is low, time reduction can only be achieved via traditional methods, such as changing the methods of completion modes. However, if the number of flexible dependencies and the rate of supplementary tasks increase, then the role of flexible approaches becomes more valuable. However, this study does not delve into further investigations, as its main objective is to demonstrate how researchers might examine the efficacy of flexible methodologies.

Table 2 shows the descriptive statistics of the three applied datasets.

**Table 2.** Descriptive statistics of project datasets considered in the experiments.

	Number of tasks	Projects	Time	Cost	Quality	Resources	Completion modes	Flexibility rates
Generated	50, 100, 200, 500	1440	+	+	+	2, 3, 4	1-4	10-40%
Synthetic	50, 100	240	+	-	-	2, 4	4	10-40%
Real	7-437	125	+	+	-	0-12	1	10-40%

The generated dataset was created with the proposed generatepdm function in the **mfpp** package. The numbers of tasks are 50, 100, 200 and 500. The project generator follows the algorithm of Kosztyán. ^16^ This allows the time, cost, quality and resources to be specified. The synthetic project dataset of Boctor ^22^ contains 240 predefined tasks with four completion modes. This dataset does not include costs. The real dataset of Batselier and Vanhoucke ^23^ contains 125 projects; however, each project has only one completion mode. Furthermore, matrix-based databases are integrated into the work of Kosztyán and Novák. ^24^ Each project has fixed structures; therefore, with the flexible project generator (FSG) ^25^ implemented in the phase3 function in the proposed **mfpp** package, 10–40% of tasks and dependencies are selected to be flexible. In this way, we obtain four times more flexible project plans. *The experimental simulation shows how flexible project planning expands the scheduling abilities of the project manager.* If there are no flexible dependencies, the project plans cannot be restructured. The increase in the flexibility rate enables the utilization of flexible project management approaches.

**Figure 2.**
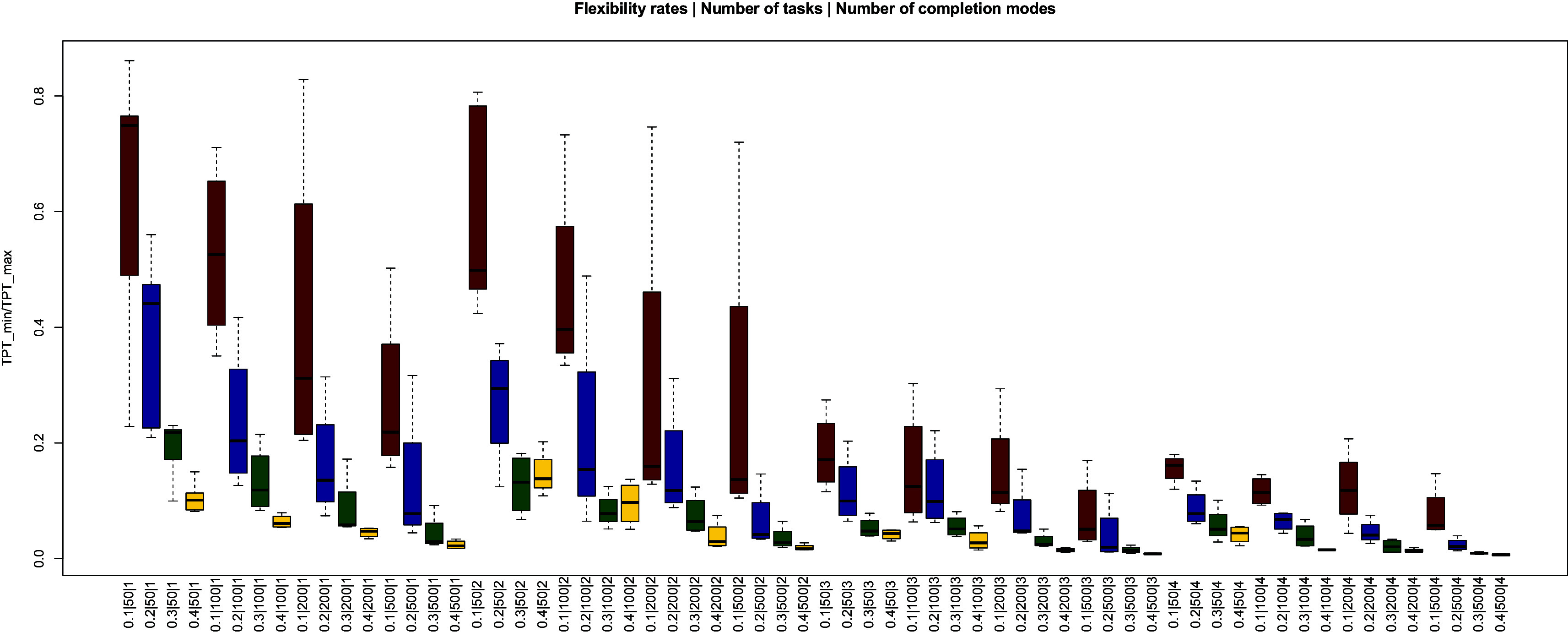
The effects of flexibility, number of tasks, and number of completion modes on the ratio of the minimal/maximal duration.

Figure 2 shows the effects of the flexibility rate, the number of tasks, and the number of completion modes on the rate of minimal/maximal project duration (TPT).

${\mathrm{TPT}}_{\min}$ / ${\mathrm{TPT}}_{\max}$ represents the possible reduction rate in the duration of the project. The project duration can be reduced even if the flexibility rate is low, as long as there are enough completion modes and the shortest duration ( *i.e.*, the shortest technology) can be selected for each task (see TPMa). However, Figure 2 shows that the greatest reduction in the duration of the project occurs if the flexibility rate is increased.

Table 3 shows the results of the analysis of variance (ANOVA).

**Table 3.** Table of analysis of variance results for the generated project plans.

Variables	Df	Sum Sq	Mean Sq	F value	Pr ( >F )	Sig.	*R* ^2^%
Flexibility rates	1	2.161	2.1608	151.835	<2e−16	***	53.27
Completion modes	1	1.233	1.2331	86.644	<2e−16	***	30.24
Numbers of tasks	1	0.536	0.5363	37.684	3.15e-09	***	13.84
Numbers of resources	1	0.111	11.13	7.818	0.00557	**	2.64
Residuals	5756	3.643	0.0142				

(Significance codes: ‘***’: 0.001 ‘**’: 0.01)

The adjusted R squared value is 0.5185. $R^{2}\%$ shows the importance of the variables. The variable of greatest importance to the possible project duration is the flexibility rate, which follows the completion mode. Although the number of tasks and the number of resources also significantly affect the reduction in project duration, they have much less impact.

Similar results can be observed for Boctor’s ^22^ synthetic data (see Table 4(a)) and Batselier and Vanhoucke’s ^23^ real project data (see Table 4(b)).

**Table 4.** Table of analysis of variances for real project plans.

(a) Summary of the ANOVA table for Boctor’s ^22^ synthetic project plans (adjusted R squared value: 0.5300)							
Variables	Df	Sum Sq	Mean Sq	F value	Pr( >F )	Sig.	*R* ^2^%
Flexibility rates	1	1.4480	1.4480	936.983	<2e−16	***	86.40
Numbers of tasks	1	0.2276	0.2276	147.298	<2e−16	***	13.58
Numbers of resources	1	0.0004	0.0004	0.251	0.616		0.02
Residuals	956	1.4774	0.15				

(b) Summary of the ANOVA table for Bates et al.’s ^26^ real project plans (adjusted R square: 0.1168)							
Variables	Df	Sum Sq	Mean Sq	F value	Pr( >F )	Sig.	*R* ^2^%
Flexibility rates	1	2.575	2.5748	44.969	5.47e-11	***	65.20
Numbers of tasks	1	0.082	0.0819	1.431	0.232		1.45
Numbers of resources	1	1.292	1.2922	22.569	2.66e-06	***	33.35
Residuals	496	28.399	0.0573				

(Significance codes: ‘***’: 0.001; ‘**’: 0.01; ‘*’: 0.05; ‘.’: 0.1; ‘ ’: 1)

In the case of the synthetic and real project databases, the completion modes were the same in all cases, so their effects are not shown in Table 4. In the case of real projects, only one completion mode was defined, so if the flexibility rate was low, only traditional approaches could be used; however, if the flexibility rate was high, agile approaches could be used. For the synthetic project database, four completion modes were defined, so the effects of traditional and hybrid approaches could be compared in these cases. In all the cases, the importance of the flexibility rate had the greatest impact on the possible reduction in project duration.

The proposed mfpp package can also be used to conduct additional investigations regarding costs and resource requirements.

## Software description

**mfpp** is a new R package for project managers to use to schedule traditional and flexible project plans. It compares different project management approaches with respect to their scheduling performance and risk mitigation to help decision makers choose the best project management approach. Additionally, when analyzing project libraries, scholars can analyze various project management approaches and their resilience to risks. This package uses existing R packages **Matrix,** ^26^ **pracma** ^15^ and **Rfast** ^27^ to generate the PDM and calculate project values; **genalg** ^28^ and **nsga2R** ^29^ for optimized resource allocation; and **ggplot2** ^30^ and **igraph** ^31^ to plot the project structure. The **mfpp** package is available for download from CRAN and Code Ocean. ^32^ A summary of the functions incorporated into this package is provided in Table 5.

**Table 5.** Summary of functions in the mfpp package.

Function	Description
generatepdm	Generates the project domain matrix (PDM) for a flexible project planning problem
get.structures	Calculates the minimal/maximal/most likely project structures
is.flexible	Checks the flexibility of the project data matrix
maxscore_PEM	Calculates the maximal score value (PMAX) of possible project scenarios
minscore_PEM	Calculates the minimal score value of possible project scenarios
paretores	Calculates the Pareto-optimal resource allocation
percent	Calculates the desired project completion characteristics of a project structure
phase1	Simulates estimation uncertainty
phase2	Simulates shock effects
phase3	Simulates the effects of a change in customer claims
plot.mfpp	Plotting function for matrix-based flexible project planning
summary.mfpp	Prints project data matrix constraints, matrices, lists, sets, etc.
tpc	Calculates the cost demands of a project
tpq	Calculates the total project quality for a project structure
tpr	Calculates the maximum resource demands of a project
tpt	Evaluates the activity times(early start time, early finish time, late start time and late finish time) of a project
truncpdm	Function for dropping excluded tasks

### Databases related to project management

Two project databases are directly included in this package. The first is the ^22^ database, which contains 240 simulated projects, all of which include four completion modes. This database can be used to test algorithms for the multimode resource-constrained project scheduling problem (MM-RC-PSP). The second database is provided by ^23^ and contains 125 real projects. These projects each include only one completion mode, but they also include the cost demands of the tasks.

## Use cases

The implementation of the R package **mfpp** is demonstrated via (i) simulated project structures and (ii) a real-life dataset of project structures. The package must be loaded at the beginning of the session by typing the following:

install.packages("mfpp") *# Install mfpp package, if it is required*
library(mfpp)


### Simulated project structures

**Build fixed matrix-based projects and calculate their demands to support TPMa**

We start with the *tpt* function of the **mfpp** package to build fixed matrix-based projects and calculate their demands to support the TPMa. The LD specifies the structure of the project. The LD is an N-by-N (sub)matrix, where N is the number of tasks. The diagonal values represent the task priorities for task completion, where a value of 1 indicates a mandatory task, and a lower value indicates a supplementary task. The off-diagonal values represent the dependencies between tasks, where a value of 1 indicates a fixed dependency, and lower values indicate flexible dependencies between tasks. In the case of an acyclic graph, the LD can be reordered as an upper triangular matrix, which is assumed in this package. In the following example, a binary logic plan with one completion mode is specified. The *tpt* function determines the duration of the project (total project time, TPT) by calculating the task schedule, including time specifications such as early start time (EST), early finish time (EFT), late start time (LST), late finish time (LFT), scheduled start time (SST), and scheduled finish time (SFT) for each task. The plot function draws a Gantt chart for ESTs, LSTs, or SSTs. The LD and TD must be specified as necessary and sufficient arguments for this function. The output of the function is as follows:

LD<-rbind(c(1,0,1), c(0,1,0), c(0,0,1))
colnames(LD)<-rownames(LD)<-paste("a",1:3,sep = "")
TD<-c(3,4,5)
TPT<-tpt(LD,TD)
summary(TPT)

##
##
##  Table of schedule
##    Dur EST EFT LST LFT TF SST SFT SF Is.Crit
## a1   3   0   3   0   3  0   0   3  0    TRUE
## a2   4   0   4   4   8  4   0   4  4   FALSE
## a3   5   3   8   3   8  0   3   8  0    TRUE

SST <- c(1,1,0)
TPT<-tpt(LD,TD,SST)
summary(TPT)

##
##
##  Table of schedule
##    Dur EST EFT LST LFT TF SST SFT SF Is.Crit
## a1   3   0   3   0   3  0   1   4 -1    TRUE
## a2   4   0   4   4   8  4   1   5  3   FALSE
## a3   5   3   8   3   8  0   4   9 -1    TRUE


The plot of the schedule can be drawn as follows ( Figure 1(a)):

Next, we illustrate the use of the *tpc*, *tpq* and *tpr* functions to calculate the total project cost, the total project quality, and the total project resources. The *tpr* function calculates the maximal resource demands and can specify a resource graph. In these functions, the CD and QD are N-by-w matrices, whereas the RD is an N-by- $\left(w^{*}r\right)$ matrix, where r is the number of resources. The CD is mandatory, whereas the QD and RD are optional matrices. The necessary arguments of these functions are the LD, CD, quality parameters (q), the QD with completion modes, and the RD. The output of this function for the given example is as follows:

set.seed(6)
CD<-c(10,20,24)
cat("\nTotal Project Cost (TPC): ", tpc(LD,CD))

##
## Total Project Cost (TPC):     54

q<-runif(3)
cat("\nTotal Project Quality (TPQ): ", tpq(LD,LD,q))

##
## Total Project Quality (TPQ):     0.5316529

QD2<-cbind(q,runif(3)) *# Generate two completion modes*
cat("\nRelative TPQ: ",tpq(LD,LD,q,QD2))

##
## Relative TPQ:     0.7169994

RD<-round(cbind(runif(3,min = 0,max=5),runif(3,min=0,max=5)))
cat("\n\nTotal Project Resources (TPR)\n\n")

##
##
## Total Project Resources (TPR)

tpr(TPT$SST,LD,TD,RD)

##     R_1 R_2
## TPR   9   8


**Figure 3.**
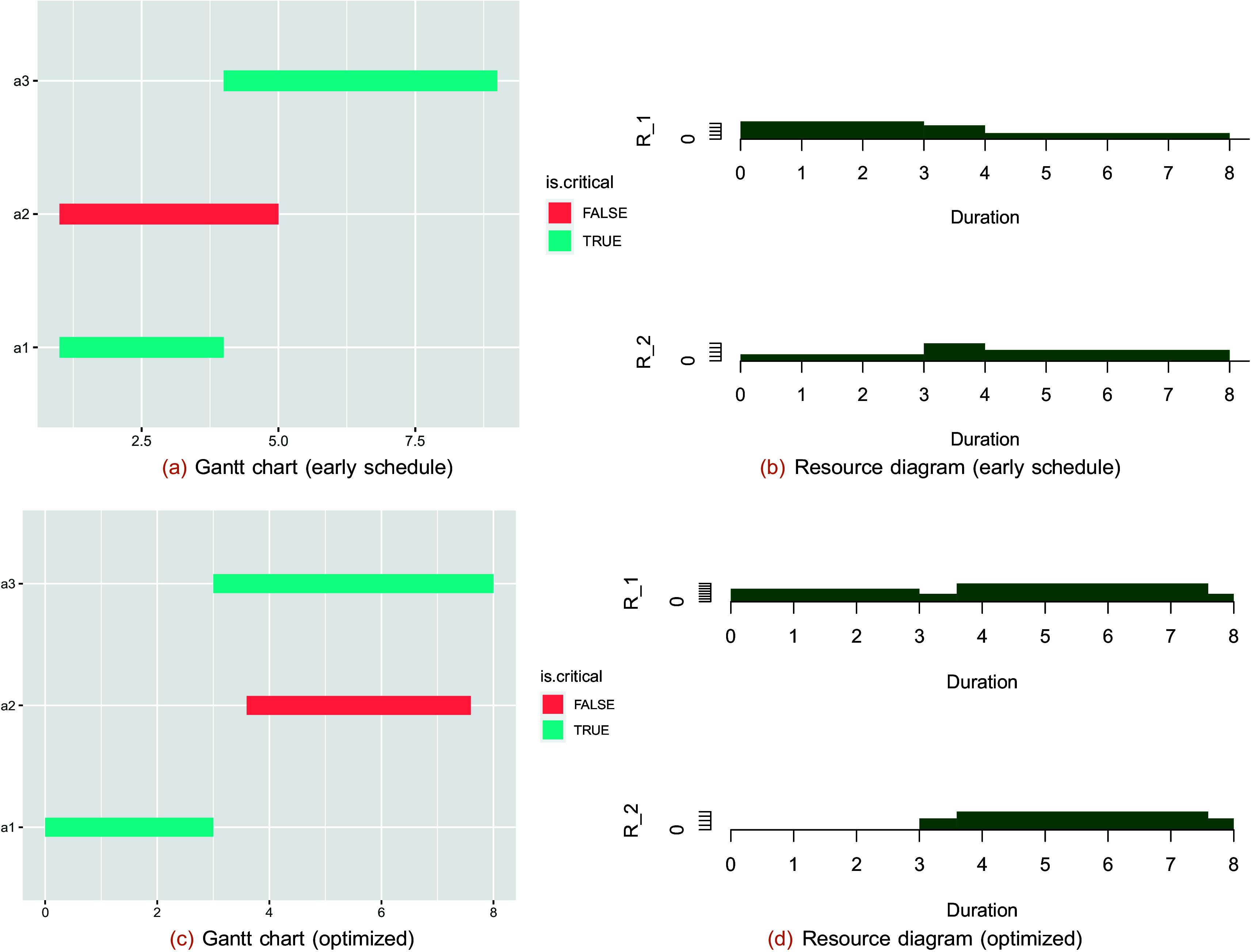
The scheduled Gantt chart and resource diagram for a binary logic plan with one completion mode. The total project resources are based on two resource domains ( $R_{1}$ and $R_{2}$ ) and are extracted by the mfpp package.

A plot of the total project resources can be displayed with the additional argument “res.graph=TRUE”, and the output is shown in Figure 3(b).

tpr(TPT$SST,LD,TD, res.graph = TRUE)

##     R_1 R_2
## TPR   9   8


Optimizing resource allocation is a combinatorial (NP-hard) problem; therefore, a metaheuristic method is used to minimize the maximum resource demands. In this case, the nondominated sorting genetic algorithm II (NSGA-II) method is used to minimize the maximum resource demands. In the case of multiple resources, a Pareto-optimal solution is found. The *paretores* function can help a project engineer determine the minimal resource demands while maintaining a specified project duration. The output of this function is shown in Figure 3(c) and in Figure 3(d).

The set of domains, namely, the set of the LD, TD and CD and optionally the QD and RD, specifies the PDM. A logic network can be constructed using this set of domains as follows:

PDM<-cbind(LD,TD,CD,q,RD)
class(PDM)<-"PDM_matrix"
summary(PDM,w=1,Rs=2)

##
## summary PDM matrix:
##    a1 a2 a3 TD CD         q
## a1  1  0  1  3 10 0.6062683 5 0
## a2  0  1  0  4 20 0.9376420 4 3
## a3  0  0  1  5 24 0.2643521 3 5
## attr(,"class")
## [1] "PDM_matrix"
##
## Minimal constraints:
##
## Summary of the PDM constraints structure:
##
## Time constraint (Ct):  8
## Const constraint (Cc):  54
## Score/scope constraint (Cs):  1
## Quality constraint (Cq):  0.5316529
##
## Maximal constraints:
##
## Summary of the PDM constraints structure:
##
## Time constraint (Ct): 8
## Const constraint (Cc): 54
## Score/scope constraint (Cs): 1
## Quality constraint (Cq): 0.5316529
## Resource constraint(s) (CR):

##     R_1 R_2
## TPR   9   8


**Generation of a flexible project network to support the HPMa**

The HPMa combines features of the TPMa (multiple completion modes), APMa (a flexible project structure), and XPMa (new, unplanned tasks). Project plans that support the HPMa can also be designed using the proposed **mfpp** package. For this purpose, the first function needed is *generatepdm.* The input arguments of this function are the number of tasks (N); the flexibility factor (ff); the connectivity factor (cf); the maximum values of the TD (mTD), CD (mCD), and RD (mRD); the number of modes (w); the number of resources (nR); the number of possible extra tasks (nW); and the scale and QD of the project scenario. The function returns either the PDM only or a PDM list that also contains the number of completion modes (w) and the number of resources (Rs). Consider a flexible plan with 4 planned tasks, 2 completion modes and 2 resources. The project schedule generated via the HPMa is as follows:

*# Generation of a PDM for flexible project planning with the MFPP package.*
*# Define the number of modes, flexibility factor and connectivity factor of a project scenario.*
N=5;ff=0.30;cf=0

*# Define the maximum values of the time domain, cost domain and resource domain of a project scenario.*
mTD=3;mCD=4;mRD=3

*# Define the number of modes, number of resources, number of possible extra tasks (nW), scale and*
*# quality domain of a project scenario.*

w=2;nR=2;nW=1
scale=1.6
PDM<-generatepdm(N,ff,cf,mTD,mCD,mRD,w,nR,nW, scale,QD=TRUE,lst=TRUE)
rownames(PDM$PDM)<-colnames(PDM$PDM)[1:(N+nW)]<-paste("a",1:(N+nW),sep="")

summary(PDM) *# Summary of PDM list*

##
## Summary of the PDM list:
##
## Number of completion modes (w): 2
## Number of resources (Rs): 2
## Summary PDM:
##   a1        a2        a3        a4 a5 a6       t_1      t_2       c_1
## a1 1 0.6348251 0.0000000 1.0000000  0  0 1.8922066 2.206381 2.9384623
## a2 0 1.0000000 1.0000000 0.0000000  0  0 0.1397774 0.160757 0.5917513
## a3 0 0.0000000 0.9458257 0.0000000  0  0 2.7119461 2.784497 3.1012793
## a4 0 0.0000000 0.0000000 0.7420972  0  0 1.8202474 1.896929 0.5478731
## a5 0 0.0000000 0.0000000 0.0000000  1  0 2.6490032 2.922064 2.3434704
## a6 0 0.0000000 0.0000000 0.0000000  0  0 0.0000000 0.000000 0.0000000
##          c_2       q_1       q_2     r_1.1    r_2.1     r_1.2     r_2.2
## a1 2.9456676 0.4591163 0.4597432 1.7602166 1.767857 0.7749634 0.8299197
## a2 0.7051308 0.2476199 0.2898660 0.9645262 1.109172 1.9847076 2.0104963
## a3 3.8735180 0.3979600 0.4327975 2.3338386 2.723688 2.3193125 2.3997095
## a4 0.6072272 0.2989701 0.3343502 1.8691629 2.192587 2.5611961 2.9716221
## a5 2.5560046 0.4891549 0.5480301 2.2484773 2.280209 2.2384662 2.4961772
## a6 0.0000000 0.0000000 0.0000000 0.0000000 0.000000 0.0000000 0.0000000
## attr(,"class")
## [1] "PDM_matrix"
##
## Minimal constraints:
##
## Summary of the PDM constraint structure:
##
## Time constraint (Ct):   2.711946
## Const constraint (Cc):   5.873684
## Score/scope constraint (Cs):   0.4907664
## Quality constraint (Cq):   0.1039251
##
## Maximal constraints:
##
## Summary of the PDM constraint structure:
##
## Time constraint (Ct):   2.945254
## Const constraint (Cc):   10.68755
## Score/scope constraint (Cs):   0.9427112
## Quality constraint (Cq):   0.3830448
## Resource constraint(s) (CR):

##          R_1      R_2
## TPR 7.196484 7.867509


The main components of flexible project structures can be shown with the *plot* function of the **mfpp** package; these structures are defined as follows.

**Minimal structure:** This structure contains only mandatory tasks and fixed dependencies. It provides the lowest quality, cost, and time demands when the lowest quality, lowest cost, and shortest completion modes are specified for each task.

**Maximal structure:** This structure includes mandatory and supplementary tasks as well as fixed and flexible dependencies. It provides the highest quality, greatest cost, and greatest time demands when the highest quality, greatest cost, and longest completion modes are specified for each task.

**Minimax structure:** This structure contains only mandatory tasks but both fixed and flexible dependencies. It can provide the lowest resource demands.

**Maximin structure:** This structure contains both mandatory and supplementary tasks but only fixed dependencies. It can provide the greatest resource demands.

**Figure 4.**
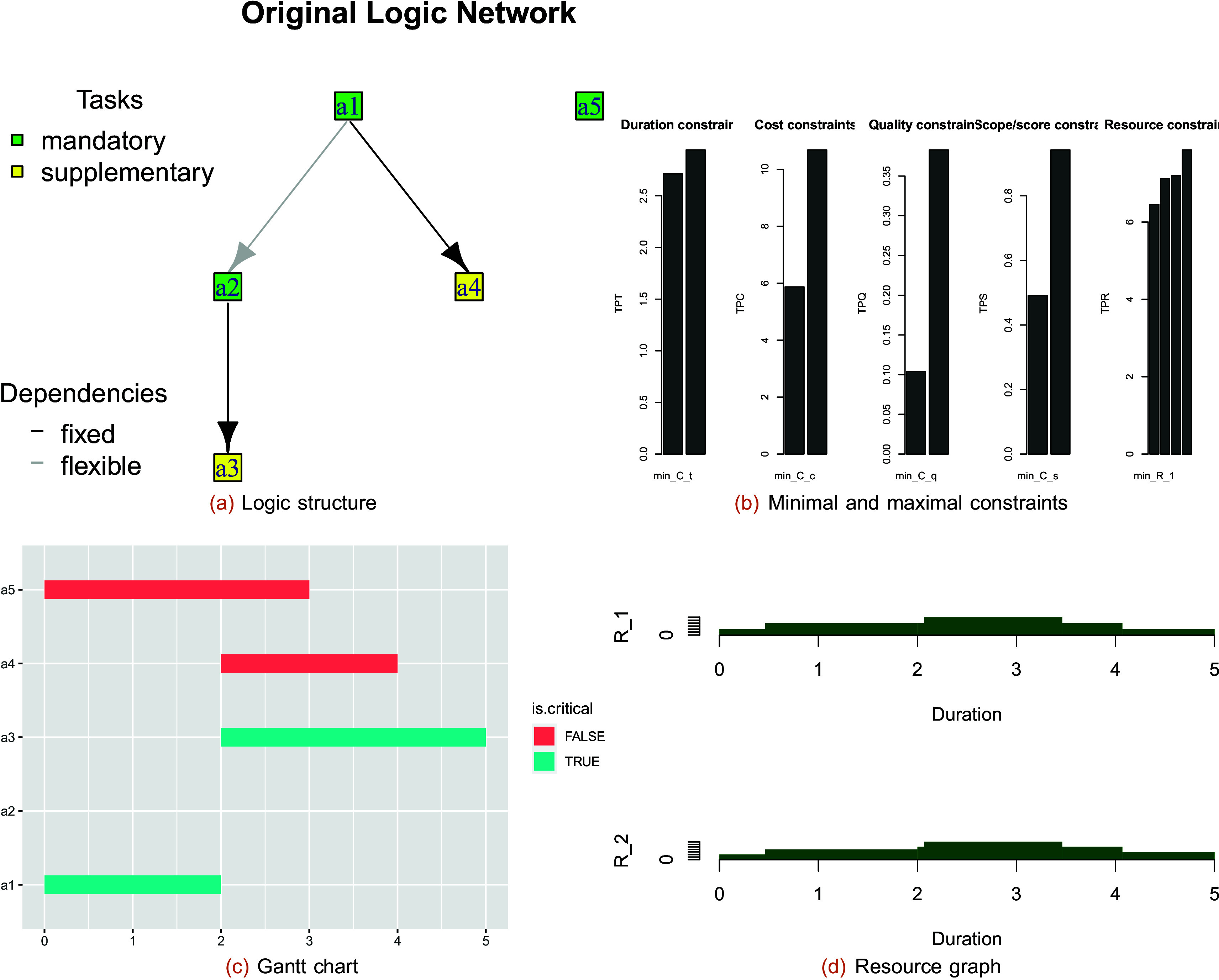
Logic structure (a) and minimal and maximal constraints (b) of the hybrid project; Gantt chart (c) and resource graph (d) of the optimal schedule for completion mode 1.

**Most likely structure:** Rounding the values of the LD yields the most likely structure. The plot of the PDM structure is shown in Figure 4(a) below.

The plot of the constraints is as follows in Figure 2(b). The most likely/most desired structures of the project for multiple modes can be determined using **mfpp**. The input and output are as follows:

PSM_list<-get.structures(PDM,type = "most")$moststruct
summary(PSM_list)

##
## summary PDM list:
##
## Number of completion modes (w): 2
## Number of resources (Rs): 2
## summary PDM matrix:
##   a1 a2 a3 a4 a5 a6       t_1     t_2        c_1       c_2       q_1       q_2
## a1 1  1  0  1  0  0 1.8922066 2.206381 2.9384623 2.9456676 0.4591163 0.4597432
## a2 0  1  1  0  0  0 0.1397774 0.160757 0.5917513 0.7051308 0.2476199 0.2898660
## a3 0  0  1  0  0  0 2.7119461 2.784497 3.1012793 3.8735180 0.3979600 0.4327975
## a4 0  0  0  1  0  0 1.8202474 1.896929 0.5478731 0.6072272 0.2989701 0.3343502
## a5 0  0  0  0  1  0 2.6490032 2.922064 2.3434704 2.5560046 0.4891549 0.5480301
## a6 0  0  0  0  0  0 0.0000000 0.000000 0.0000000 0.0000000 0.0000000 0.0000000
##        r_1.1    r_2.1     r_1.2     r_2.2
## a1 1.7602166 1.767857 0.7749634 0.8299197
## a2 0.9645262 1.109172 1.9847076 2.0104963
## a3 2.3338386 2.723688 2.3193125 2.3997095
## a4 1.8691629 2.192587 2.5611961 2.9716221
## a5 2.2484773 2.280209 2.2384662 2.4961772
## a6 0.0000000 0.000000 0.0000000 0.0000000
## attr(,"class")
## [1] "PDM_matrix"
##
## Minimal constraints:
##
## Summary of the PDM constraints structure:
##
## Time constraint (Ct):  2.851723
## Const constraint (Cc):  9.522836
## Score/scope constraint (Cs):  1
## Quality constraint (Cq):  0.3665425
##
## Maximal constraints:
##
## Summary of the PDM constraints structure:
##
## Time constraint (Ct):  2.945254
## Const constraint (Cc):  10.68755
## Score/scope constraint (Cs):  1
## Quality constraint (Cq):  0.4025323
## Resource constraint(s) (CR):

##          R_1      R_2
## TPR 7.196484 7.867509


The optimal schedules for different completion modes can be calculated via the *paretores* and *truncpdm* functions by dropping excluded tasks and their demands. The optimal resource allocation can be calculated after the completion modes are selected for each task, as follows:

*# Get PSM*
*# Drop excluded tasks and their demands*
PSM<-round(truncpdm(PSM_list$PDM))

w<-PSM_list$w *# Get number of completion modes*
Rs<-PSM_list$Rs *# Get number of resources*

DSM<-PSM[,1:nrow(PSM)] *# Get PSM*

*# Get time demands (time domain, TD)*
TD<-PSM[,paste("t",1:w,sep = "_")]

*# Get resource demands (resource domain, RD)*
RD<-PSM[,tail(colnames(PSM),w*Rs)]

*# Calculate optimal schedules for both completion modes*

RES1<-paretores(DSM,TD[,1],RD[,1:Rs])
RES2<-paretores(DSM,TD[,2],RD[,(Rs+1):(2*Rs)])


Figure 4(b) shows the Gantt chart, and Figure 4(c) shows the resource graph of the optimal schedule for completion mode 1.

## Project structure database

In this section, Boctor’s publicly available ^22^ simulation database is used to demonstrate the applicability of **mfpp**. This database contains a collection of 240 projects with different names, numbers of completion modes (w) and numbers of resources (Rs). The details of the database can be retrieved via the summary function. However, we use **project number 2** from this database, the details of which are as follows: name of project= boct10, w= 4 and Rs= 2. Any other project could also be used to obtain the optimal project structure and resources.

### Obtaining a project structure from the collection

**Figure 5.**
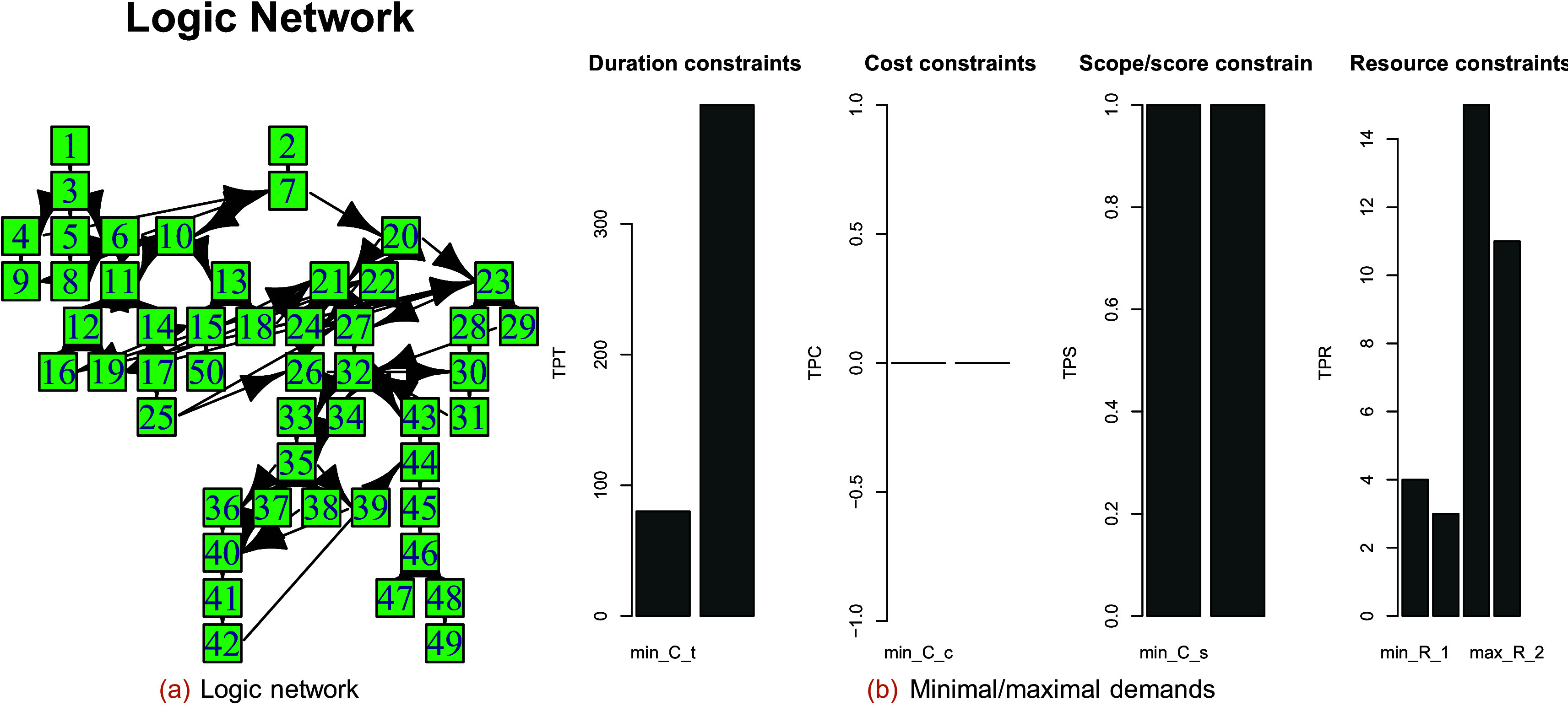
Logic network and minimal/maximal demands of the project with completion mode 4 in the Boctor database.

Figure 5(a) shows the original project structure, and Figure 5(b) shows the minimal/maximal constraints of project 2 from the Ref. 22 project collection.

**Figure 6.**
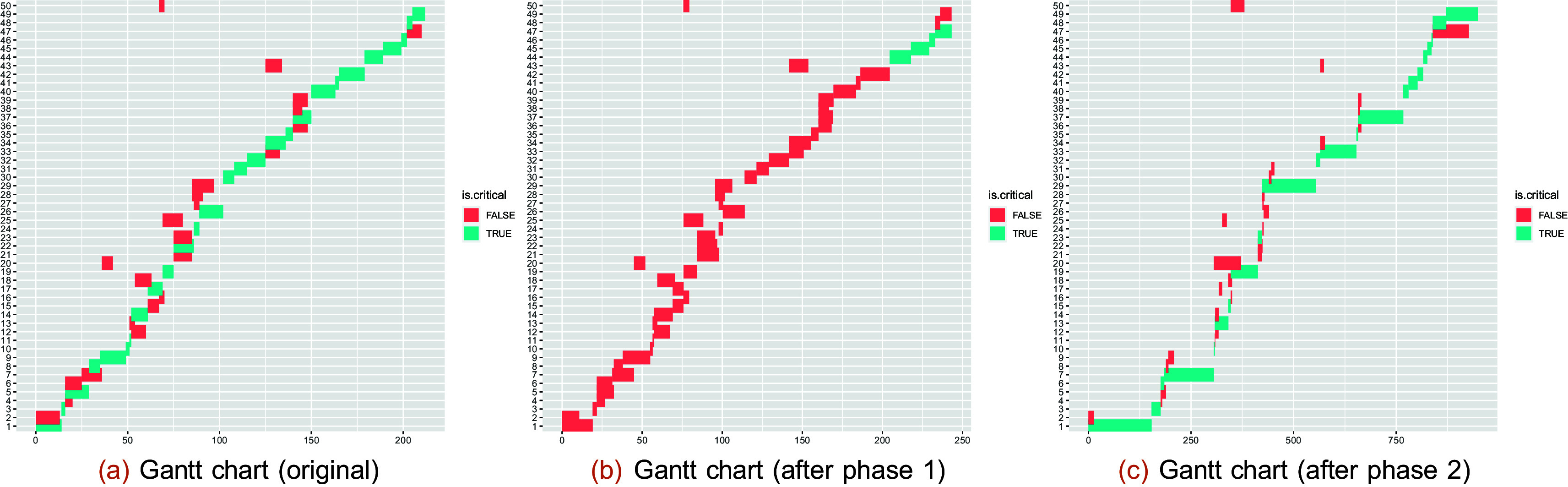
Gantt chart of the selected project for the 1st completion mode scheduled by early starting time (a); after varying project demands (b-c) (see phase 1 and phase 2).

The Gantt charts of the selected project for completion mode 1 are constructed by extracting the PDM and TD as follows (see Figure 6(a)):

### Sensitivity analysis

One of the most frequently used methods of project risk analysis is sensitivity analysis. The sensitivity analysis procedure in **mfpp** has three phases. **Phase 1:** Effects of uncertainty **Phase 2:** Effects of shocks **Phase 3:** Structural changes.

Notably, phase 1 and phase 2 involve varying demands, whereas phase 3 varies the project structure. Phase 1 affects the entire project, while phases 2 and 3 affect only selected demands or structural elements. These phases can be applied in a sequential or parallel manner. The phases follow the logic of Ref. 5. The **mfpp** package also analyzes the effects of uncertainty on the scheduled project via the *phase1* and *phase2* functions. Furthermore, the existing project plan can be modified to a flexible structure via the *phase3* function in the developed package. These functions are also demonstrated on the project selected from the database.

**Effects of uncertainty**

Phase 1 analyzes the uncertainty of the estimation. Modified demands are generated in the interval ([o+a,o+b]) , where o is the original value. The random generator can be specified to follow either a *uniform* (default) or *beta* distribution. These modifications are applied to all types of demands for each task (compare Figures 6(a), 6(b), and 6(c)).

**Effects of shocks**

Phase 2 simulates shock effects by increasing p percent of the task demands by a factor of up to s . Phase 2 investigates the effects of shocks, where not all task demands are changed but the changes are significant (see Figure 6(b)).

**Structural changes**

**Figure 7.**
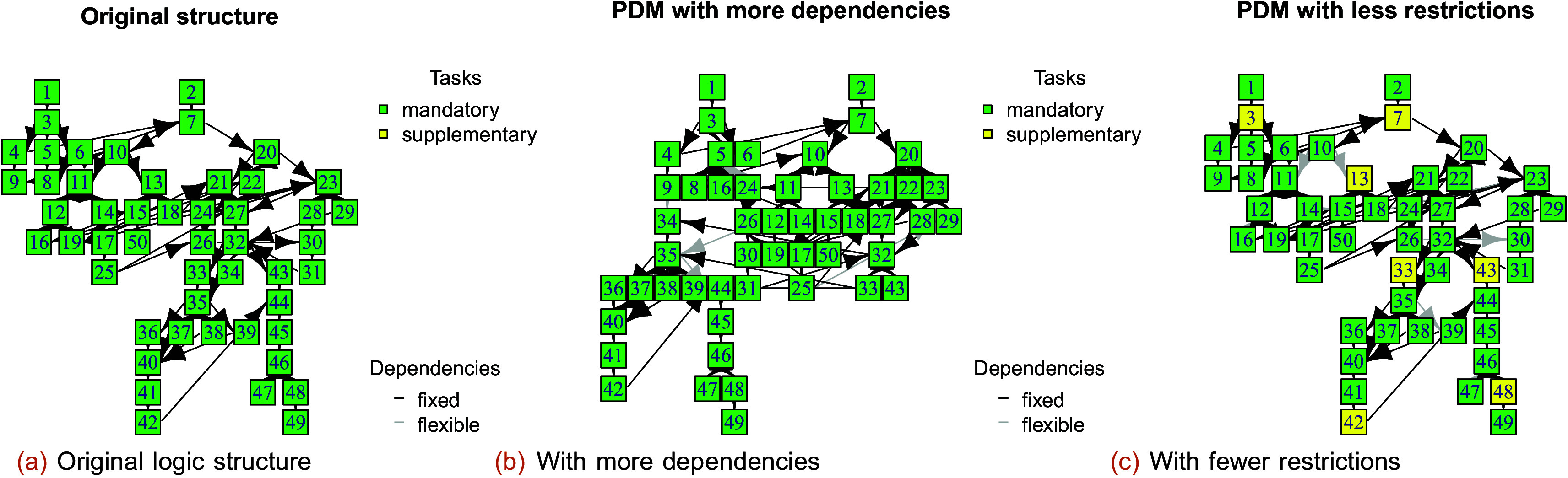
Logic network of the original structure (a) with more dependencies (b) and with fewer restrictions (c) (see the phase 3 function).

In phase 3, P percent of the nodes ( *i.e.,* tasks) or arcs ( *i.e.,* dependencies) are selected to change the corresponding scores by up to the maximal change effect S . Phase 3 simulates changes in customer priorities and technological changes. In the case of S<0 , the plan will be more flexible, whereas S>0 increases priorities and can produce new dependencies (compare Figures 7(a), 7(b), and 7(c)).

## Limitations and future work

To our knowledge, mfpp is the only package in R that can handle flexible dependencies and completion priorities of tasks; however, this package could be further developed in many ways. First, each dependency between tasks is considered a finish-to-start dependency. Although this is the most common dependency in practice, the implementation of additional possible dependencies would provide further application and research potential. Kosztyán and Novák ^24^ converted many more project databases into a matrix-based form. The integration of these databases into the mfpp package would offer further possibilities for investigation. The NSGA2 algorithm is used for optimization; however, another metaheuristic method could be employed to find optimal solutions.

## Conclusions

The **mfpp** package was developed to provide users with a comprehensive set of functions that can be used to create matrix-based models for both traditional and flexible project management approaches. The presented package also compares different project management approaches regarding their scheduling performance and risk mitigation to help decision-makers choose the best project management approach. Moreover, when analyzing project libraries, scholars can analyze various project management approaches and their resilience to risks. The **mfpp** tool can help decision-makers determine which approaches are best for their requirements.

## Data Availability

The mfpp package includes matrix-based versions of the publicly available Boctor ^22^ and Batseliers ^23^ project datasets.

## Software availability

**Source code available at:** https://codeocean.com/capsule/5196188/tree/v1

**Archived source code at the time of publication:** https://doi.org/10.24433/CO.2915306.v1 ^32^

**License:** MIT license

mfpp can be installed via CRAN with install.packages(“mfpp”).

mfpp is maintained at http://github.com/kzst/mfpp.
