# Supplementary material for: {MFPP(R). An R package for matrix-based flexible project planning
Source: F1000Res. 2024 Sep 12;13:356. Originally published 2024 Apr 23. [Version 2] doi: 10.12688/f1000research.143144.2 (PMC12619900; doi:10.12688/f1000research.143144.2)
Supplement: Peer review history

# Reviewer report: Reviewer response for version 2

**DOI:** 10.5256/f1000research.170545.r418367

**Published:** 2025-11-15

**Authors:** Marzieh Aghileh

**Affiliations:** 1 University of Minho, Guimarães, Portugal

- Update references to recent literature
- Increase the number of references
- Several references are authored by Kosztyán and co-authors (2015, 2018, 2020, 2023). While relevant, the paper would be stronger if it included more independent validation from other researchers.
- Currently only a few limitations are listed. Add discussion on: Data availability and the difficulty of validating with real-world projects.

Are the conclusions about the tool and its performance adequately supported by the findings presented in the article?

Yes

Is the rationale for developing the new software tool clearly explained?

Yes

Is the description of the software tool technically sound?

Yes

Are sufficient details of the code, methods and analysis (if applicable) provided to allow replication of the software development and its use by others?

Yes

Is sufficient information provided to allow interpretation of the expected output datasets and any results generated using the tool?

Yes

Reviewer Expertise:

Project scheduling, Resource constraint, Project Management, Uncertainty, Resource Flexibility

I confirm that I have read this submission and believe that I have an appropriate level of expertise to confirm that it is of an acceptable scientific standard.

# Reviewer report: Reviewer response for version 2

**DOI:** 10.5256/f1000research.170545.r323048

**Published:** 2025-11-15

**Authors:** Bálint Blaskovics

**Affiliations:** 1 Corvinus University of Budapest, Budapest, Hungary

The authors did an excellent job to address my comments. Now I think both academics and practitioners could benefit from this paper (especially those who are operating in engineering). All in all, I'm satisfied and I'd like to thank you for the opporunity to review this.

Are the conclusions about the tool and its performance adequately supported by the findings presented in the article?

Partly

Is the rationale for developing the new software tool clearly explained?

Partly

Is the description of the software tool technically sound?

Yes

Are sufficient details of the code, methods and analysis (if applicable) provided to allow replication of the software development and its use by others?

Yes

Is sufficient information provided to allow interpretation of the expected output datasets and any results generated using the tool?

Partly

Reviewer Expertise:

project management

I confirm that I have read this submission and believe that I have an appropriate level of expertise to confirm that it is of an acceptable scientific standard.

# Reviewer report: Reviewer response for version 1

**DOI:** 10.5256/f1000research.156774.r292541

**Published:** 2024-07-05

**Authors:** Bálint Blaskovics

**Affiliations:** 1 Corvinus University of Budapest, Budapest, Hungary

The paper addresses an interesting topic, namely how can we apply R for project planning for non-predictive projects as well. The authors start with a brief and focused introduction, however, I would welcome a bit more about time plotting techniques, and non-predictive project management approaches to provide the necessary background for the next sections (or at least about the different, non-deterministic scheduling techniques). I would also welcome more about either the research gap or the comparison of the different software packages in order to evaluate the results more. I would also dedicate a bit more time to how this can be beneficial for future studies and/or the use case of programming in this way.

 All in all, I think it is a well-based, scientific paper, however, I would welcome a bit more changes in order to be consumable for everyone who is interested in the topic.

Are the conclusions about the tool and its performance adequately supported by the findings presented in the article?

Partly

Is the rationale for developing the new software tool clearly explained?

Partly

Is the description of the software tool technically sound?

Yes

Are sufficient details of the code, methods and analysis (if applicable) provided to allow replication of the software development and its use by others?

Yes

Is sufficient information provided to allow interpretation of the expected output datasets and any results generated using the tool?

Partly

Reviewer Expertise:

project management

I confirm that I have read this submission and believe that I have an appropriate level of expertise to confirm that it is of an acceptable scientific standard, however I have significant reservations, as outlined above.

# Response

**Published:** 2024-08-19

**Authors:** Zsolt Tibor Kosztyán

**Affiliations:** Department of Quantitative Met, University of Pannonia, Egyetem str. 10, Veszprém, Hungary

Dear Dr. Bálint Blaskovics,

 Thank you for the valuable remarks. According to your remarks and those of the other reviewers, we have thoroughly revised the manuscript. All the remarks were addressed. The responses are shown in red. In the revised manuscript, insertions are shown in blue, and deletions are shown in red.

 Sincerely,

 The Authors

 Q.1: The paper addresses an interesting topic, namely how can we apply R for project planning for non-predictive projects as well. The authors start with a brief and focused introduction, however, I would welcome a bit more about time plotting techniques, and non-predictive project management approaches to provide the necessary background for the next sections (or at least about the different, non-deterministic scheduling techniques). I would also welcome more about either the research gap or the comparison of the different software packages in order to evaluate the results more. I would also dedicate a bit more time to how this can be beneficial for future studies and/or the use case of programming in this way.

 All in all, I think it is a well-based, scientific paper, however, I would welcome a bit more changes in order to be consumable for everyone who is interested in the topic.

**Response: **The paper has been fully revised. Nondeterministic techniques are briefly introduced. However, most nondeterministic scheduling techniques, such as discrete, continuous and stochastic time‒cost tradeoff methods, where more than one technology is used or where the exact time demands are not known, can be considered flexible; therefore, they are detailed along with the other considered types of flexibility. Our contributions to the literature are stated, and the limitations of our methods are also specified.

# Reviewer report: Reviewer response for version 1

**DOI:** 10.5256/f1000research.156774.r284888

**Published:** 2024-07-04

**Authors:** Ágoston Kolos

**Affiliations:** 1 Corvinus University, Budapest, Hungary

I expect that the methodology used is not well known to R users in general. Therefore, a short but precise mathematical formulation of the models (TPMa, APMa, XPMa, HPMa) is needed. I think the authors can do this in the appendix or provide an external article or link. In the latter case, it is important to give a reference to another source where the description of the model is clear and precise (not a general overview).

 There are typos in the user manual ("Sumulated project database")

Are the conclusions about the tool and its performance adequately supported by the findings presented in the article?

Yes

Is the rationale for developing the new software tool clearly explained?

Partly

Is the description of the software tool technically sound?

Yes

Are sufficient details of the code, methods and analysis (if applicable) provided to allow replication of the software development and its use by others?

Partly

Is sufficient information provided to allow interpretation of the expected output datasets and any results generated using the tool?

Yes

Reviewer Expertise:

Operations research

I confirm that I have read this submission and believe that I have an appropriate level of expertise to confirm that it is of an acceptable scientific standard, however I have significant reservations, as outlined above.

# Response

**Published:** 2024-08-19

**Authors:** Zsolt Tibor Kosztyán

**Affiliations:** Department of Quantitative Met, University of Pannonia, Egyetem str. 10, Veszprém, Hungary

Dear Dr. Ágoston Kolos,

 Thank you for the valuable remarks. According to your and the other reviewers’ remarks, we have thoroughly revised the manuscript. All the remarks were addressed. The responses are shown in red. In the revised manuscript, insertions are shown in blue, and deletions are shown in red.

 Sincerely,

 The Authors

 Q.1: I expect that the methodology used is not well known to R users in general. Therefore, a short but precise mathematical formulation of the models (TPMa, APMa, XPMa, HPMa) is needed. I think the authors can do this in the appendix or provide an external article or link. In the latter case, it is important to give a reference to another source where the description of the model is clear and precise (not a general overview).

**Response:** The paper has been fully revised. We present the problem and refer to a publication containing the details.

# Reviewer report: Reviewer response for version 1

**DOI:** 10.5256/f1000research.156774.r292539

**Published:** 2024-06-29

**Authors:** Shan Jayasinghe

**Affiliations:** 1 Southampton Solent University, Southampton, England, UK

Thank you so much for the opportunity to review the paper.

 The paper makes the assertion that "while project planning and scheduling tools are already available to support traditional project management approaches, flexible project management, such as agile, extreme, and hybrid project planning, are only somewhat supported by computer algorithms." This claim is not accurate. Advanced project management tools such as JIRA, Asana, and Monday.com are backed by highly sophisticated algorithms and are widely used to manage adaptable projects across numerous multinational companies.

 The first paragraph does a satisfactory job of comparing traditional and adaptive project management approaches. However, the research gap of the paper is inadequately defined and needs to be more clearly established to strengthen the overall argument and relevance of the study.

 Apart from the above concern, the following improvements must be made to enhance the paper to a indexable level.

 -Include more recent studies and compare the proposed method with state-of-the-art project management tools.

 -Organize the methodology section with clear headings and subheadings. Use more diagrams to illustrate key concepts.

 -Conduct a more detailed performance evaluation of the mfpp package, comparing it with existing tools and discussing its scalability.

 -Include a discussion on the limitations of the proposed approach and potential areas for future work.

 -Ensure all references are complete and correctly formatted.

 -Ensure the manuscript is written in clear, concise language with appropriate academic tone and style. Revise for grammar, punctuation, and clarity to enhance readability and coherence.

Are the conclusions about the tool and its performance adequately supported by the findings presented in the article?

No

Is the rationale for developing the new software tool clearly explained?

No

Is the description of the software tool technically sound?

No

Are sufficient details of the code, methods and analysis (if applicable) provided to allow replication of the software development and its use by others?

No

Is sufficient information provided to allow interpretation of the expected output datasets and any results generated using the tool?

No

Reviewer Expertise:

project management, technology adoption, teaching and learning

I confirm that I have read this submission and believe that I have an appropriate level of expertise to state that I do not consider it to be of an acceptable scientific standard, for reasons outlined above.

# Response

**Published:** 2024-08-19

**Authors:** Zsolt Tibor Kosztyán

**Affiliations:** Department of Quantitative Met, University of Pannonia, Egyetem str. 10, Veszprém, Hungary

Dear Reviewer Dr. Shan Jayasinghe,

 Thank you for the valuable remarks. According to your remarks and those of the other reviewers, we have thoroughly revised the manuscript. We hope that the academic standards of the revised manuscript have been improved to meet your expectations. All the remarks were addressed. The responses are shown in red. In the revised manuscript, insertions are shown in blue, and deletions are shown in red.

 Sincerely,

 The Authors

 Thank you so much for the opportunity to review the paper.

 Q.1: The paper makes the assertion that "while project planning and scheduling tools are already available to support traditional project management approaches, flexible project management, such as agile, extreme, and hybrid project planning, are only somewhat supported by computer algorithms." This claim is not accurate. Advanced project management tools such as JIRA, Asana, and Monday.com are backed by highly sophisticated algorithms and are widely used to manage adaptable projects across numerous multinational companies.

**Response:** Thank you for the comment. We have clarified this statement both in the abstract and the introduction. You are correct. There are a few commercial project management software programs, such as JIRA, Asana, Wrike, Teamwork, Zoho Projects, and Monday.com, which support flexible project planning. The target audience of these software tools is professional users and not scholars. These software applications are not freely available. The algorithms for managing flexible projects in these applications are hidden from users. In general, they provide only the most likely solution and not all possible solutions, in contrast to the algorithms used here (Kosztyán-Szalkai 2018). Only with an implementation such as ours can the smallest and largest resource requirements and the smallest and largest possible time and cost demands be estimated.

 In addition, in R software, there is no package that is backed by sophisticated algorithms and can handle flexible project management, such as agile, extreme, or hybrid project planning.

 Q.2: The first paragraph does a satisfactory job of comparing traditional and adaptive project management approaches. However, the research gap of the paper is inadequately defined and needs to be more clearly established to strengthen the overall argument and relevance of the study.

**Response:** The first part of the paper has been fully revised. The contributions of the paper are stated. The background of the method has been made more detailed.

 Q.3: Include more recent studies and compare the proposed method with state-of-the-art project management tools.

**Response:** The revised paper refers to more recent studies and details the benefits of the proposed package.

 Q.4:Organize the methodology section with clear headings and subheadings. Use more diagrams to illustrate key concepts.

**Response:** The methodology section “Brief summary of matrix-based project planning and risk analysis” has been added and revised. The new figure (Figure 1) compares the matrix-based plans of different types of project management approaches.

 Q.5: Conduct a more detailed performance evaluation of the mfpp package, comparing it with existing tools and discussing its scalability.

**Response:** A new Experiments section has been provided to show the performance and possibilities of the mfpp packages. The main purpose of this section was to show researchers that such tests can be performed by presenting the proposed package. During the generation process, we also examined projects containing 500 activities. We allowed several execution methods and considered several types of resource requirements. The results are included in the Experiments section. Since existing tools cannot calculate all possible alternatives, the possible time reduction cannot be calculated.

 Q.6: Include a discussion on the limitations of the proposed approach and potential areas for future work.

**Response:** In the revised paper, a new Limitations and Future Works section has been added.

 Q.7:Ensure all references are complete and correctly formatted.

**Response:** Thank you. This has been corrected.

 Q.8: Ensure the manuscript is written in clear, concise language with appropriate academic tone and style. Revise for grammar, punctuation, and clarity to enhance readability and coherence.

**Response: **Thank you. The final version was proofread by American Journal Experts.

**References:**

****

**Kosztyán, Z. T., & Szalkai, I. (2018). Hybrid time-quality-cost trade-off problems. *Operations Research Perspectives*, *5*, 306-318.**
